# Nano‐Backpack Engineered Probiotics with Reactive Oxygen Species–Responsive Tungsten Release for Synergistic Therapy of Inflammatory Bowel Disease

**DOI:** 10.1002/advs.76291

**Published:** 2026-06-26

**Authors:** Yang Yang, Liucan Wang, Guoqing Chen, Yuanling Zhang, Jiarui Shi, Wenzhe Fan, Min Yu, Jixi Zhang, Hua Yang

**Affiliations:** ^1^ Chongqing Medical University Chongqing China; ^2^ Department of General Surgery Chongqing Academy of Medical Sciences Chongqing General Hospital Chongqing University Chongqing China; ^3^ Key Laboratory of Biorheological Science and Technology College of Bioengineering Ministry of Education Chongqing University Chongqing China

**Keywords:** composite probiotic system, inflammatory bowel disease, microecological regulation, responsive release, selective antibacterial

## Abstract

Integrating probiotics with functional nanomaterials to disrupt the cycle of gut microbiota dysbiosis and reactive oxygen species (ROS) overload is an emerging therapeutic strategy for inflammatory bowel disease (IBD). However, simplistic combinations of therapeutics often result in spatiotemporal mismatch within the gastrointestinal tract, preventing precise intervention at target inflammatory sites. To address this, a composite probiotic system, BSCS@WO_3_@PDA is developed, centered around programmed ROS‐mediated cascade regulation for intelligent synergistic therapeutic intervention. The system is constructed by electrostatic assembly of polydopamine (PDA)‐coated tungsten trioxide nanoparticles onto chitosan‐encapsulated *Bacillus subtilis* (BS), thereby significantly enhancing gastrointestinal stability and promoting prolonged retention in the inflamed colon. At the inflammatory site, the ROS‐responsive PDA shell enabled on‐demand ROS scavenging while undergoing degradation, leading to the sustained release of tungsten ions. These ions specifically inhibited molybdenum cofactor‐dependent nitrate reductase activity in Enterobacteriaceae metabolism, thereby creating a favorable ecological niche for probiotics. Consequently, the retained BS promoted beneficial microbes and metabolites, reversing colitis‐associated metabolic disorders and synergistically enhancing intestinal barrier repair. This study proposes a novel paradigm of precise cascade metabolic regulation by integrating ROS scavenging and pathogen inhibition. The BSCS@WO_3_@PDA system offers a powerful new strategy for restoring gut microecological homeostasis and treating inflammatory diseases.

## Graphical abstract

An integrated probiotic system is engineered to execute a programmed therapeutic cascade against inflammatory bowel disease. Upon pathological signals, the system sequentially performs ROS scavenging, tungsten release, and selective pathogen suppression, thereby breaking the oxidative stress–dysbiosis cycle to remodel gut microbiota and reinforce mucosal barrier integrity. This strategy offers a novel platform for the modulation of gut microecology.

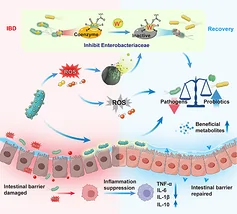

## 1 Introduction

Inflammatory bowel disease (IBD), characterized by chronic intestinal inflammation and gut microbiota dysbiosis, poses a significant global health challenge due to its increasing incidence and prevalence [1]. The primary pathological mechanism involves a vicious cycle between oxidative stress and pathogenic microbiota expansion. Specifically, inflammatory conditions trigger immune cells to release excessive reactive oxygen species (ROS), which damage the intestinal epithelium and amplify inflammatory signaling [2] while altering the intestinal redox potential. This shift promotes the abnormal proliferation of facultative anaerobic pathogens such as Enterobacteriaceae [3, 4]. These pathogens utilize inflammation‐derived electron acceptors like nitrate for respiration, inhibit probiotics through nutrient competition and biofilm formation, and exacerbate intestinal epithelial barrier damage and immune dysregulation [5]. Current treatments primarily utilize broad‐spectrum antibiotics and immunosuppressants; however, prolonged use can disrupt microbiota diversity, induce drug resistance, and elevate infection risks [6, 7]. Therefore, there is an urgent need for novel IBD therapies that concurrently address oxidative stress, selectively target pathogenic microorganisms, and restore intestinal microbiota homeostasis.

Probiotic‐based therapy has emerged as a promising strategy for the prevention and treatment of colitis. Evidence has shown that probiotics can exert therapeutic effects by modulating the gut microbiota, promoting the production of beneficial metabolites such as butanoic acid, and enhancing intestinal barrier function [8]. Compared to conventional pharmacological treatments, oral probiotics offer multiple advantages, including fewer adverse effects and improved patient compliance [9]. Nonetheless, the survival and function of commonly used probiotics such as anaerobic strains *Lactobacillus* and *Bifidobacterium* are significantly compromised by elevated ROS levels in the inflamed colonic microenvironment [10]. *Bacillus subtilis* (BS), an FDA‐approved probiotic and facultative anaerobe, shows potential for IBD treatment by consuming intestinal oxygen to create a hypoxic environment for other probiotics and secreting antimicrobial peptides and surfactin to inhibit the growth of pathogenic bacteria [11, 12]. However, as with other probiotics, the oral delivery of live BS faces significant challenges such as gastric acid degradation and niche competition from pathogenic Enterobacteriaceae, which compromise intestinal colonization and therapeutic efficacy [13, 14]. Strategies to enhance probiotic delivery have evolved from traditional chitosan (CS)–alginate coatings [15] and microencapsulation techniques [16] to advanced functionalization platforms. Recently, highly advanced designs, including nanoencapsulation systems responsive to the microenvironment [17, 18], degradable nanoarmors [19], and bioactive systems capable of modulating the microenvironment [20, 21], have been developed to improve bacterial survival and local delivery. Despite these advances, most current approaches still lack a single platform that integrates highly efficient ROS scavenging capacity with selective pathogen inhibition, which often restricts the effective modulation of intestinal microecology at severe inflammatory lesions.

During persistent intestinal inflammation, Enterobacteriaceae drive rapid proliferation primarily by exploiting nitrate as an electron acceptor for anaerobic respiration. Tungsten ions (W^6+^) can selectively disrupt the molybdenum (Mo^6+^) cofactor‐dependent nitrate respiration pathway in these bacteria. This disruption exclusively inhibits inflammation‐associated Enterobacteriaceae while sparing the normal commensal flora, which suppresses pathogenic bacterial overgrowth, thus offering a novel strategy for highly selective microbiota regulation [22, 23]. However, the non‐specific distribution and rapid clearance of free tungsten ions often lead to insufficient colonic concentration, limiting their therapeutic efficacy [24, 25]. Oral administration of tungsten oxide nanoparticles (WO_3_ NPs) has been shown to enhance tungsten accumulation in the colonic mucus layer [26], yet premature tungsten ion release in acidic environments may cause severe off‐target damage [25]. Due to their open mesoporous structure, mesoporous polydopamine (MPDA) particles loaded with tungsten ions for the delivery of *Lactobacillus acidophilus* resulted in leakage of tungsten ions under acidic conditions [27]. Polydopamine (PDA), a biocompatible polyphenolic polymer, offers a potential solution for controlled release due to its robust adhesion and high structural stability [28, 29]. Studies have confirmed that dopamine can self‐polymerize into PDA under alkaline conditions, forming a stable coating that persists in acidic environments and serves as a physicochemical barrier [30]. Conversely, in a high‐ROS inflammatory microenvironment, the PDA coating undergoes triggered degradation [31]. This property is promising for developing a protective interface for WO_3_ NPs, preventing premature tungsten ions leakage in acidic conditions while enabling ROS‐responsive release in the inflamed colon. Additionally, the catechol groups in PDA facilitate efficient ROS scavenging via electron transfer mechanisms, potentially disrupting the inflammation‐oxidative stress cycle in IBD and establishing a low‐oxidative stress microenvironment conducive to probiotic delivery [32, 33, 34].

**SCHEME 1:**
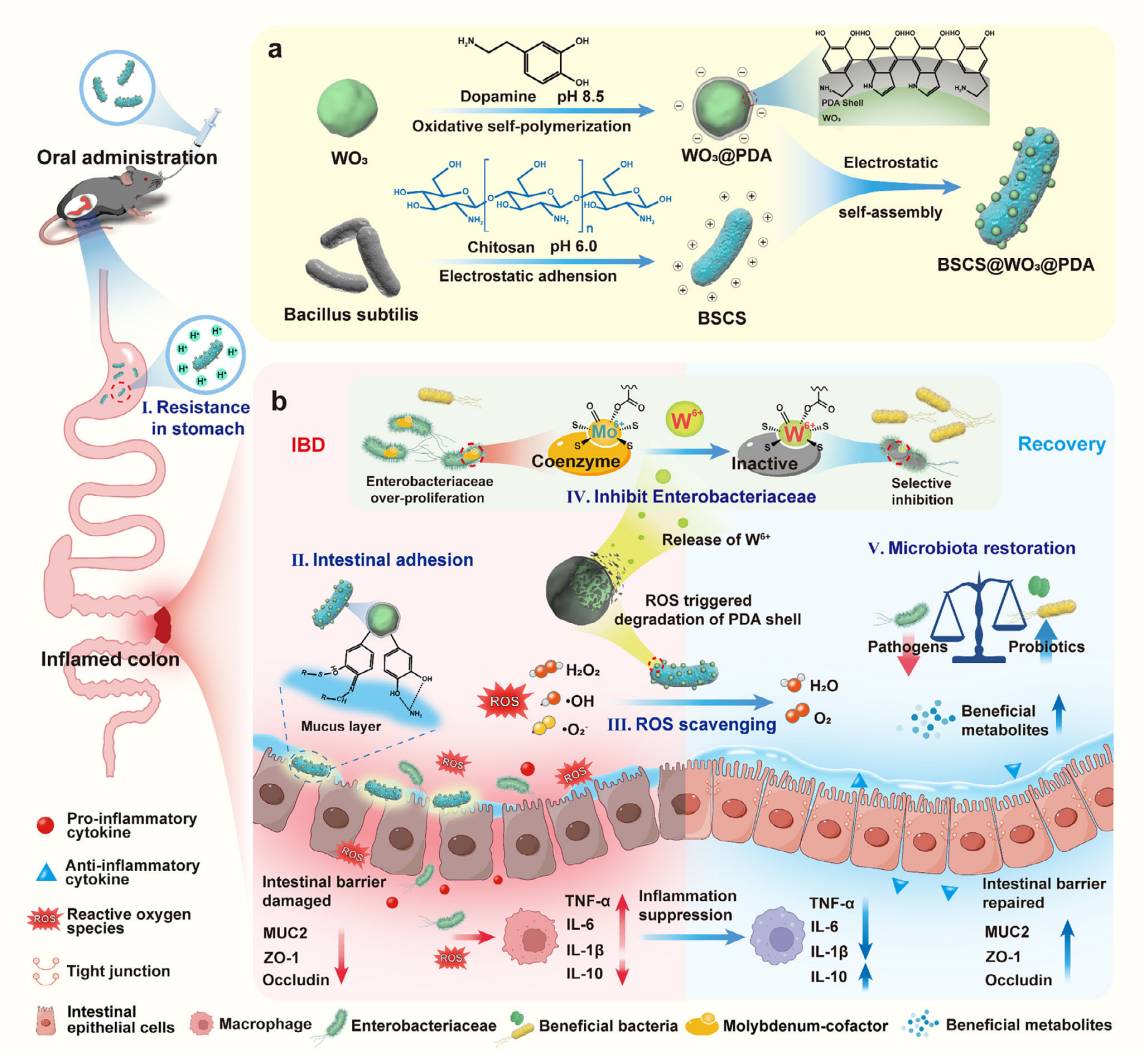
Fabrication of BSCS@WO_3_@PDA and its therapeutic mechanism for IBD. (a) Synthesis of PDA‐coated WO_3_ NPs (WO_3_@PDA NPs) via oxidative self‐polymerization of dopamine on WO_3_ NPs, followed by electrostatic assembly of WO_3_@PDA NPs onto CS‐encapsulated BS (BSCS). (b) Upon oral administration, BSCS@WO_3_@PDA protects probiotics during gastrointestinal transit. In the colon, the system scavenges ROS via the polyphenol groups in the PDA shell and inhibits pathogenic Enterobacteriaceae through tungsten ion‐mediated suppression of nitrate reductase. This programmed cascade attenuates inflammatory responses, restores gut microbiota homeostasis and metabolic function, and promotes intestinal barrier repair.

Herein, we developed a polyphenol‐modified nanoparticle‐assisted composite probiotic system designed to disrupt the vicious cycle between oxidative stress and Enterobacteriaceae over‐proliferation in IBD through precise ROS‐mediated cascade regulation. Specifically, WO_3_ NPs were encapsulated with a PDA coating to maintain their structural integrity in the gastric environment and prevent premature tungsten release. Probiotic BS was encapsulated with CS to enhance gastric acid resistance and surface potential, thereby facilitating the electrostatic assembly of the two functional components into an integrated therapeutic platform: BSCS@WO_3_@PDA (Scheme 1). In the inflammatory microenvironment, the PDA shell exerts a dual function: scavenging local ROS and undergoing responsive degradation to achieve controlled tungsten release. The liberated tungsten ions selectively inhibit the molybdenum cofactor‐dependent nitrate reductase of Enterobacteriaceae, disrupting their anaerobic energy metabolism and reconfiguring the ecological niche previously occupied by pathogenic bacteria to facilitate probiotic persistence and microbiota remodeling. Meanwhile, working in synergy with the protected BS, this targeted intervention facilitates comprehensive microecological regulation and restores intestinal homeostasis. In vitro simulations were employed to validate acid stability, ROS‐responsive degradation, and selective antibacterial efficacy. Systemic biosafety and colonic accumulation were performed to demonstrate the advantage of controlled release in mitigating off‐target toxicity. Finally, this programmed therapeutic cascade that sequentially modulates oxidative stress, pathogen expansion, and metabolic dysregulation was further studied in vivo to evaluate its potential as a synergistic therapy through targeted metabolic interventions for IBD.

## 2 Results and Discussion

### 2.1 Antioxidant, Selective Antibacterial, and Gastrointestinal Protective Properties of the Synthesized WO_3_@PDA NPs

**FIGURE 1:**
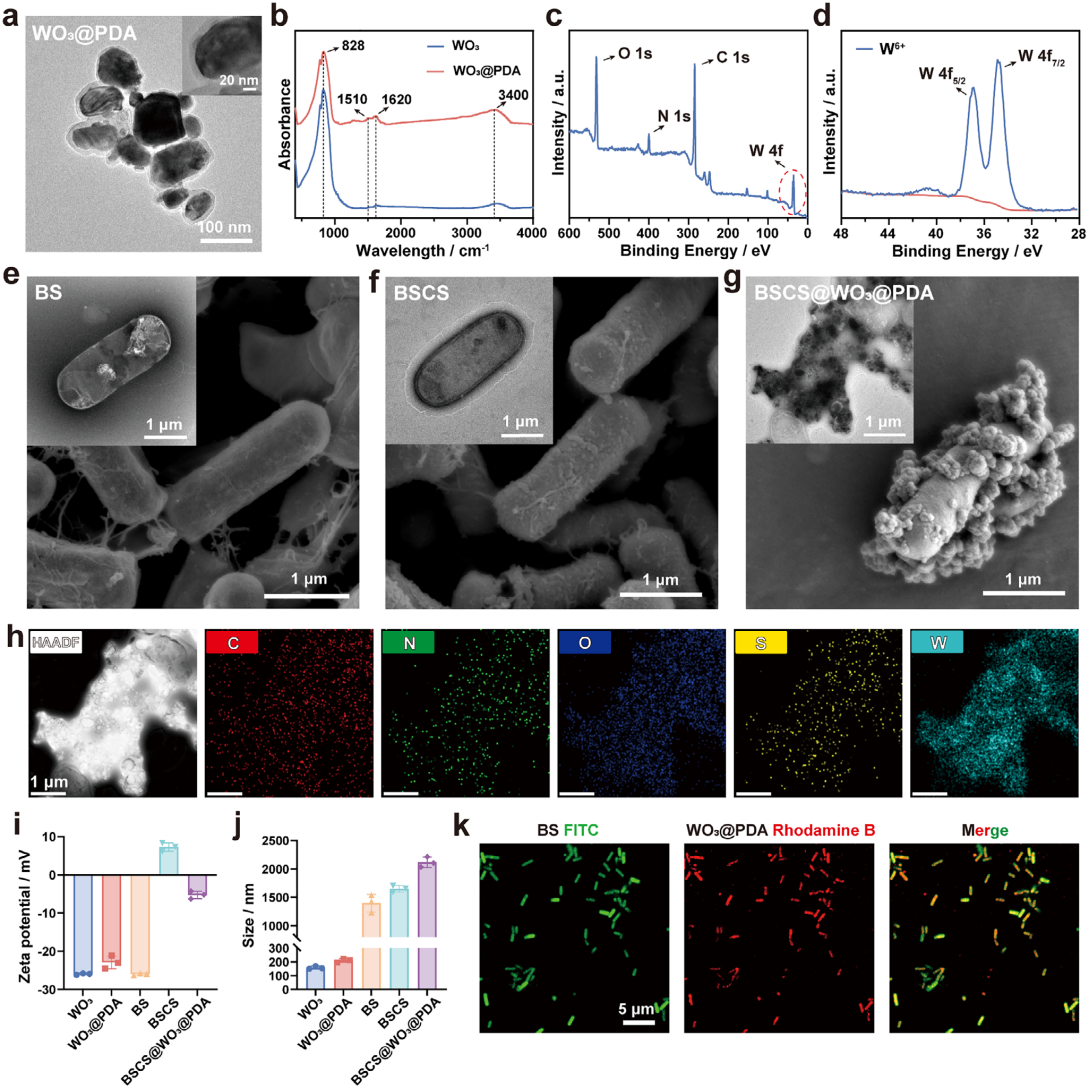
Characterization of WO_3_@PDA NPs and BSCS@WO_3_@PDA. (a) TEM image of WO_3_@PDA NPs. Scale bar: 100 nm. (b) FT‐IR spectrum of WO_3_ NPs and WO_3_@PDA NPs. (c) XPS survey spectrum of WO_3_@PDA NPs. (d) High‐resolution W 4f XPS spectrum. SEM and TEM images of (e) BS, (f) BSCS, and (g) BSCS@WO_3_@PDA. Scale bar: 1 µm. (h) HAADF‐STEM and corresponding elemental mapping images of BSCS@WO_3_@PDA (C, N, O, S, W). Scale bar: 1 µm. (i) Zeta potential and (j) hydrodynamic size distribution of components measured by DLS. (k) Confocal laser scanning microscopy (CLSM) images of BSCS@WO_3_@PDA: fluorescein isothiocyanate (FITC)‐labeled BS (green) and Rhodamine B‐labeled WO_3_@PDA NPs (red). Scale bar: 5 µm.

WO_3_@PDA NPs were synthesized through aqueous oxidative polymerization [35, 36]. Digital photographs showed that WO_3_ NPs appear milky white, while WO_3_@PDA NPs appear black due to PDA modification (Figure S1). Transmission electron microscopy (TEM) images (Figure 1a) showed that the WO_3_@PDA NPs had an irregular quasi‐spherical shape with an average diameter of approximately 100 nm. In contrast to pristine WO_3_ NPs (Figure S2), a distinct PDA shell approximately 10 nm thick was observed. Dynamic light scattering (DLS) confirmed an increase in the hydrodynamic diameter of WO_3_@PDA NPs relative to WO_3_ NPs (Table S1 and Figure S3). Elemental mapping demonstrated the uniform distribution of W, C, N, and O elements within the WO_3_@PDA NPs (Figure S4). Fourier transform infrared (FT‐IR) spectroscopy (Figure 1b) indicated that pristine WO_3_ NPs had a major absorption peak at 828 cm^−1^, attributed to O‐W‐O stretching vibrations [37]. In contrast, WO_3_@PDA NPs exhibited characteristic PDA peaks at 1620 cm^−1^ (aromatic C = C vibration) and 1510 cm^−1^ (C‐N‐H deformation vibration of indole), along with a broad absorption peak at 3450 cm^−1^ (overlapping N‐H and O‐H stretching vibrations) [38]. X‐ray photoelectron spectroscopy (XPS) analysis confirmed the presence of W, C, N, and O elements in WO_3_@PDA NPs. The chemical state of tungsten was identified as W^6^^+^ based on the characteristic doublet at the binding energies of 35.40 eV (W 4f_7/2_) and 37.52 eV (W 4f_5/2_) (Figure 1c,d). These findings indicate the successful coating of WO_3_ NPs with PDA, forming a core‐shell structured nanocomposite.

Subsequently, the probiotic BS was coated with the positively charged polysaccharide CS to obtain BSCS. In contrast to the smooth surface of unmodified BS (Figure 1e), the scanning electron microscopy (SEM) images revealed a roughened, wrinkled film enveloping the bacteria, while the TEM images showed a distinct, uniform coating layer with lower electron contrast adhering to the bacterial surface (Figure 1f). Then, WO_3_@PDA NPs at various weight ratios were assembled onto the surface of BSCS via a simple yet efficient electrostatic interaction to construct the composite probiotic system BSCS@WO_3_@PDA_x_. As shown in the SEM images (Figure S5), the surface of BSCS@WO_3_@PDA_100_ (1 × 10^7^ colony‐forming units [CFU] BS / 100 µg WO_3_@PDA) was uniformly decorated with numerous sub‐spherical NPs. In contrast, higher NP concentrations led to pronounced particle aggregation. Consequently, BSCS@WO_3_@PDA_100_ was selected as the optimal formulation for all further investigations. TEM imaging (Figure 1g) revealed a high density of NPs adhering to the bacterial surface. Elemental mapping (Figure 1h) revealed the distribution of C, N, O, S, and W within the composite. The S signal indicates intrinsic bacterial components, while the distinct localization of W elements on bacterial surfaces confirms the successful loading of WO_3_@PDA NPs.

Zeta potential analysis showed that pristine WO_3_ NPs had a potential of approximately −26 ± 0.2 mV, which shifted to approximately −23 ± 1.7 mV after PDA modification. Unmodified BS exhibited a surface potential of −26 ±0.26 mV. Due to the cationic nature of CS, the potential of BSCS increased significantly to approximately +8 ± 1 mV. Upon conjugation of WO_3_@PDA NPs with BSCS, the final composite BSCS@WO_3_@PDA showed a surface potential near −5.2 ± 1 mV (Figure 1i), validating the success of the modification strategy. The stepwise increase in hydrodynamic diameter supported the system design (Figure 1j). To visually verify WO_3_@PDA NPs integration on the BS surface, BS and WO_3_@PDA NPs were labeled with FITC and Rhodamine B, respectively, and assembled into BSCS@WO_3_@PDA. CLSM revealed green fluorescent bacteria decorated with red fluorescence (Figure 1k), indicating strong particle adsorption on the bacterial surface. UV–vis absorption spectra showed no notable spectral shifts during synthesis (Figure S6). These results confirm the successful construction of BSCS@WO_3_@PDA.

**FIGURE 2:**
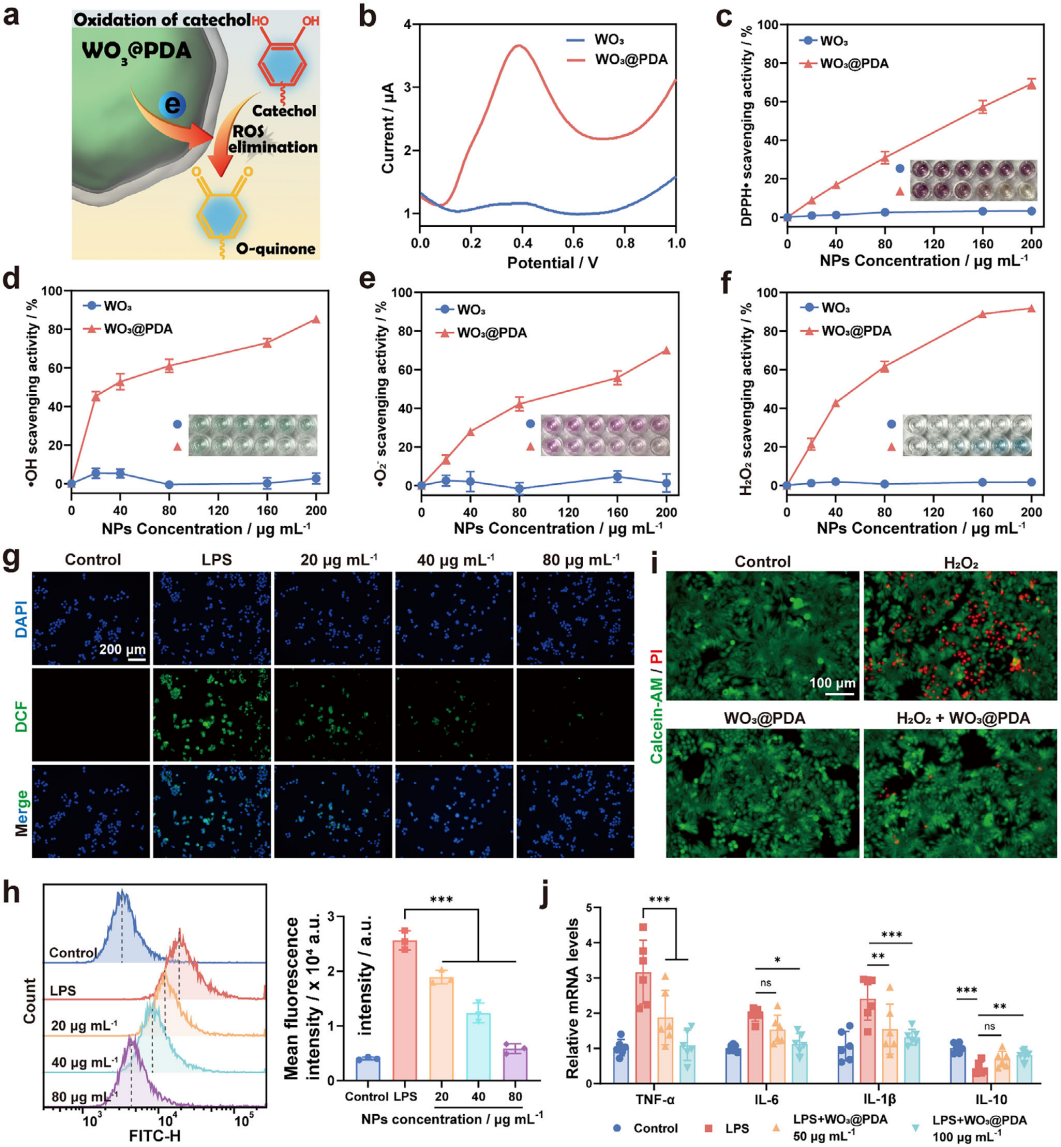
Evaluation of the in vitro antioxidant and anti‐inflammatory effects of WO_3_@PDA NPs. (a) Polyphenol‐mediated electron‐transfer antioxidant mechanism of WO_3_@PDA NPs. (b) DPV curves of WO_3_ NPs and WO_3_@PDA NPs in 0.1 M phosphate‐buffered saline (PBS). Radical scavenging evaluation of varying concentrations of WO_3_@PDA NPs toward (c) DPPH•, (d) •OH, (e) •O_2_^−^_,_ and (f) H_2_O_2_, with reactions visualized by color changes. (g) Fluorescence images of intracellular ROS scavenging by WO_3_@PDA NPs at varying concentrations in lipopolysaccharide (LPS)‐induced HT‐29 cells, detected by DCFH‐DA staining. Scale bar: 200 µm. (h) Flow cytometric quantification of DCF fluorescence intensity (*n* = 3). (i) Live/Dead (Calcein‐AM/PI) staining of HT‐29 cells under 2 mM H_2_O_2_ treatment. Scale bar: 100 µm. (j) Relative expression levels of cytokine mRNA (TNF‐α, IL‐6, IL‐1β, and IL‐10) in LPS‐induced RAW 264.7 cells after treatment with 100 ng mL^−1^ LPS and varying concentrations of WO_3_@PDA NPs (*n* = 6). Data are presented as mean ± standard deviation (SD). *p < 0.05, **p < 0.01, ***p < 0.001; ns, not significant.

The pathological progression of IBD is critically driven by the excessive accumulation of ROS. Polyphenols, recognized as potent bioactive compounds, have demonstrated significant efficacy in mitigating oxidative stress and suppressing inflammatory responses [39]. Therefore, we evaluated the broad‐spectrum ROS scavenging capability of WO_3_@PDA NPs. As illustrated in Figure 2a, the radical‐neutralizing functionality is attributed to an electron transfer mechanism mediated by catechol groups within the surface‐bound PDA layer, facilitated through phenol‐quinone conversion. Electrochemical validation via differential pulse voltammetry (DPV) revealed a distinct oxidation peak at 0.4 V (vs. Ag/AgCl) for WO_3_@PDA NPs (Figure 2b), providing direct electrochemical evidence for this mechanism.

Subsequently, the ROS scavenging capacity of WO_3_@PDA NPs (0–200 µg mL^−1^) against free radicals (DPPH•, •OH, •O_2_^−^) and H_2_O_2_ was quantified. At 200 µg mL^−1^, WO_3_@PDA NPs achieved scavenging efficiencies of 70% for DPPH•, 85% for •OH, and 70% for •O_2_^−^ (Figure 2c–e). Simultaneously, the system attained 90% H_2_O_2_ clearance, as evidenced by the inhibition of indigo carmine degradation (Figure 2f). Figure S7 illustrates the corresponding changes in UV–vis absorption peaks. In contrast, pristine WO_3_ NPs exhibited negligible activity against all ROS targets (<5% efficiency), demonstrating that PDA functionalization confers WO_3_ NPs with broad‐spectrum antioxidant functionality. Importantly, upon integration onto the bacterial surface, the ROS scavenging capacity of BSCS@WO_3_@PDA exhibited no significant alteration (Figure S8).

Building upon the exceptional in vitro ROS scavenging capability of WO_3_@PDA NPs, we evaluated their cellular antioxidant efficacy. LPS, a common component of the outer membrane of Gram‐negative bacteria such as *Escherichia coli* (*E. coli*), disrupts redox homeostasis and significantly elevates intracellular ROS levels, making it a widely used agent for inflammatory cell models [40]. In LPS‐stimulated HT‐29 cells, DCFH‐DA fluorescence imaging revealed intense green fluorescence in the LPS group, while WO_3_@PDA NPs‐treated cells exhibited a concentration‐dependent fluorescence reduction (Figure 2g). Flow cytometry quantitatively validated this ROS clearance effect (Figure 2h). Live/Dead staining demonstrated extensive cell death in the 2 mM H_2_O_2_ treatment group, whereas negligible cell mortality was observed in the WO_3_@PDA NPs treatment group (Figure 2i), confirming protection against oxidative damage.

In LPS‐induced RAW264.7 cells, RT‐qPCR analysis revealed significant downregulation of pro‐inflammatory cytokines (TNF‐α, IL‐6, IL‐1β) and upregulation of anti‐inflammatory IL‐10 mRNA expression in the WO_3_@PDA NPs‐treated group (Figure 2j). Concurrently, treatment with 100 µg mL^−1^ of WO_3_@PDA NPs did not induce significant alterations in the expression of inflammatory cytokines in normal cells (Figure S9). Collectively, these results indicate that WO_3_@PDA NPs effectively mitigate cellular oxidative stress by scavenging ROS, thereby exerting anti‐inflammatory effects.

**FIGURE 3:**
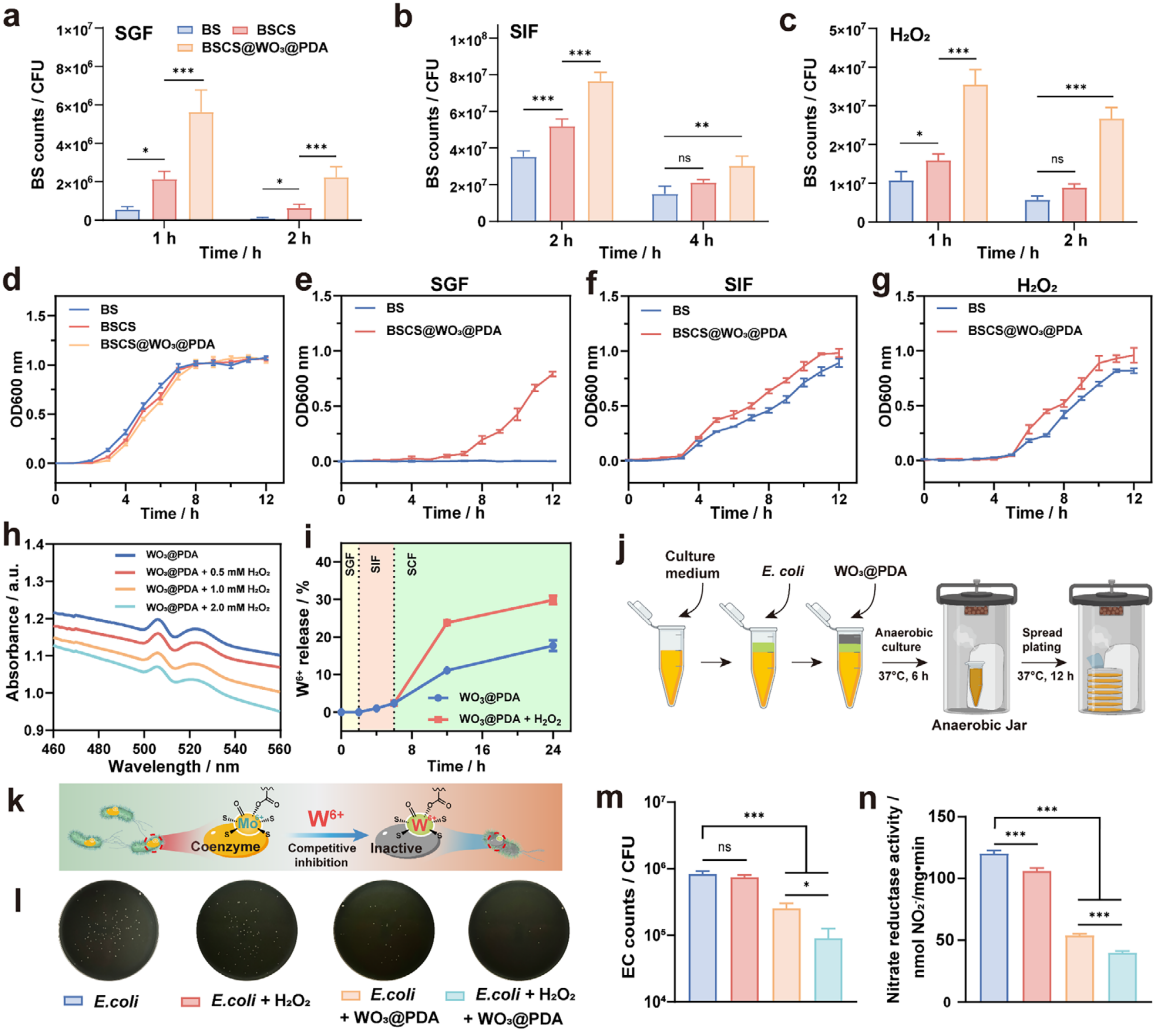
Evaluation of the gastrointestinal protective effect of WO_3_@PDA NPs on probiotics and their inhibitory activity against Enterobacteriaceae. Colony counts of plates after exposure to (a) SGF, (b) SIF, and (c) H_2_O_2_. (d) Growth curves of BS, BSCS, and BSCS@WO_3_@PDA in LB broth (monitored by OD_600_). Growth curves of BS and BSCS@WO_3_@PDA in LB broth after exposure to (e) SGF, (f) SIF, or (g) H_2_O_2_ (monitored by OD_600_). (h) UV–vis absorption spectra of WO_3_@PDA NPs at increasing H_2_O_2_ concentrations (0–2 mM). (i) Tungsten ions release profile of WO_3_@PDA NPs in simulated gastrointestinal environments measured by inductively coupled plasma mass spectrometry (ICP‐MS). (j) Schematic diagram of the experiment for assessing the inhibition of *E. coli* by WO_3_@PDA NPs. (k) Schematic diagram of the mechanism whereby W^6+^ inhibits nitrate reductase activity in Enterobacteriaceae by competitively substituting for Mo^6+^ in the molybdenum cofactor, thereby inactivating the enzyme. (l) Representative plate images, (m) colony counts, and (n) nitrate reductase activity assay of *E. coli* after anaerobic co‐incubation with WO_3_@PDA NPs. Data are presented as mean ± SD (*n* = 3). *p < 0.05, **p < 0.01, ***p < 0.001; ns, not significant.

The modification strategy was found to have no significant adverse effect on probiotic viability. First, plate counting revealed comparable CFU between BS, BSCS, and BSCS@WO_3_@PDA (Figure S10). Furthermore, although the growth curves of BSCS and BSCS@WO_3_@PDA—monitored via OD_600_—exhibited a slight initial lag compared to unmodified BS due to the physical barrier of the surface coating, both groups successfully reached a comparable stationary phase (Figure 3d), confirming that the composite system retains intrinsic bacterial activity.

Orally administered probiotics face challenges from the harsh gastrointestinal environment (e.g., gastric acid and digestive enzymes) and elevated ROS in the inflamed colonic microenvironment. To simulate oral delivery, BS, BSCS, and BSCS@WO_3_@PDA were incubated in simulated gastric fluid (SGF; pH 1.5, containing 0.32% pepsin), simulated intestinal fluid (SIF; pH 6.8, containing 10 mg mL^−1^ pancreatin) or 1 mM H_2_O_2_ in PBS at 37°C. Immediate post‐treatment survival was determined by plate counting viable bacteria at designated time points (Figure S11). As shown in Figure 3a, BS exhibited severely compromised survival in SGF, confirming that acidic conditions and pepsin induce substantial probiotic death. In contrast, CS coating alone provided moderate protection against gastric acid, while BSCS@WO_3_@PDA significantly enhanced survival rates. This improvement is likely attributed to WO_3_@PDA NPs embedded within the CS layer, which enhance the acid‐shielding efficacy. Although BS survival markedly increased in SIF compared to SGF, a progressive decline in viability occurred after 4 h incubation. BSCS@WO_3_@PDA maintained a higher viability (Figure 3b), demonstrating additional resistance to intestinal digestive stressors. In H_2_O_2_ challenge tests, the CS coating offered limited protection due to insufficient physical shielding. Notably, incorporating WO_3_@PDA NPs tripled BS survival rates (Figure 3c), primarily through catechol‐mediated H_2_O_2_ reduction by PDA. To evaluate the subsequent post‐treatment regrowth capacity, equal aliquots of the treated samples were incubated in fresh lysogeny broth (LB) medium at 37°C with 200 rpm agitation to generate growth recovery profiles; the OD_600_ was monitored hourly. SGF‐treated BS showed no significant increase in OD_600_, indicating severely impaired proliferative capacity. Conversely, BSCS@WO_3_@PDA entered the exponential growth phase after 6 h (Figure 3e), confirming preserved metabolic activity. Similarly, for SIF and H_2_O_2_ treatments, BSCS@WO_3_@PDA exhibited approximately 30% higher OD_600_ than unmodified BS at matched timepoints (Figure 3f,g). Furthermore, during a continuous sequential simulated gastrointestinal transit (SGF, SIF, and simulated colonic fluid [SCF]), the final survival rate of unmodified BS dropped to less than 1%, whereas the BSCS@WO_3_@PDA system maintained an approximately 16% survival rate (Figure S12). Collectively, the synergistic CS and WO_3_@PDA NPs modification strategy significantly enhanced probiotic viability and preserved regrowth potential during oral delivery.

Dysbiosis of the gut microbiota, particularly the overgrowth of pathogenic anaerobic Enterobacteriaceae and concomitant excessive release of bacterial endotoxins, exacerbates IBD symptoms and contributes to poor clinical prognosis [41]. Consequently, selective suppression of Enterobacteriaceae is crucial for IBD management. Recent studies indicate that tungsten ions (W^6+^) displace molybdenum (Mo^6+^) in the molybdenum cofactor, thereby inhibiting the nitrate reductase‐dependent anaerobic energy metabolism pathway in Enterobacteriaceae [22]. To evaluate the tungsten ion release capacity of WO_3_@PDA NPs, we first validated the ROS‐responsive degradability of the PDA shell. After incubating WO_3_@PDA NPs in varying concentrations of H_2_O_2_ for 24 h, concentration‐dependent material fading (Figure S13) and reduced UV absorption intensity (Figure 3h) were observed. Furthermore, TEM images of samples treated with 1 mM H_2_O_2_ revealed thinning and irregular morphology of the PDA shell (Figure S14). These results confirm the effective degradation of the PDA shell under high ROS conditions [31].

Subsequently, tungsten release profiles under simulated physiological conditions were quantified using ICP‐MS. As shown in Figure 3i, WO_3_@PDA NPs exhibited minimal tungsten ion release in SGF (<1% after 2 h) and SIF (2.4 ± 0.3% after 6 h), indicating efficient PDA‐mediated tungsten ion containment in acidic environments. Elevated pH in SCF resulted in moderate tungsten ion release (12.2 ± 0.8% at 12 h; 18.3 ± 1.4% at 24 h). Notably, under H_2_O_2_ stimulation (mimicking inflammatory ROS levels), tungsten ion release increased significantly to 23.8 ± 0.5% (12 h) and 29.8 ± 1.1% (24 h). These release characteristics demonstrate the acid tolerance and ROS‐triggered degradation properties of WO_3_@PDA NPs, laying a foundation for oral delivery.

To validate the inhibitory efficacy of WO_3_@PDA NPs against Enterobacteriaceae, *E. coli* DH5α was selected as a representative model strain due to its stable genotype and highly conserved nitrate respiration system. Following induction of nitrate reductase expression in LB medium supplemented with 40 mM NaNO_3_, *E. coli* was co‐incubated with WO_3_@PDA NPs under anaerobic conditions with H_2_O_2_ supplementation to simulate inflammatory microenvironments (Figure 3j). Figure 3k illustrates the mechanism of tungsten‐mediated inhibition of *E. coli*. Colony counting assays demonstrated that WO_3_@PDA NPs significantly suppressed the anaerobic growth of *E. coli* (Figure 3l,m). Critically, a further reduction in bacterial survival was observed in the presence of H_2_O_2_, consistent with triggered degradation of the PDA shell that accelerated tungsten ion release and enhanced antibacterial activity. To examine direct effects on the enzymatic target, dissimilatory nitrate reductase activity was quantified using a commercial assay kit. Treatment with WO_3_@PDA NPs markedly inhibited nitrate reductase activity in *E. coli* (Figure 3n). Notably, enzyme activity decreased by approximately 66% under H_2_O_2_ exposure compared to untreated controls.

Subsequently, we evaluated the bacteriostatic effect of BSCS@WO_3_@PDA under anaerobic conditions using MacConkey agar, a selective medium for cultivating Gram‐negative bacteria. The results revealed that in the presence of nitrate as a substrate, unmodified BS exhibited a moderate inhibitory capacity against *E. coli*, whereas BSCS@WO_3_@PDA demonstrated significant suppression of *E. coli* proliferation (Figure S15). Collectively, these findings demonstrate that WO_3_@PDA NPs achieve on‐demand release of bacteriostatic tungsten within high‐ROS inflammatory niches, effectively suppressing the anaerobic proliferation of Enterobacteriaceae and impairing their colonization advantage in the gut ecosystem.

### 2.2 Colon Accumulation and Anti‐Inflammatory Effects of BSCS@WO_3_@PDA

The biosafety profile of the materials was systematically evaluated. First, the hemocompatibility of BS, WO_3_@PDA NPs, and BSCS@WO_3_@PDA was evaluated by a hemolysis assay. The results showed negligible hemolytic activity (<2%) across all tested concentrations, confirming excellent blood compatibility (Figure S16). Subsequently, potential cytotoxicity was assessed by co‐culturing HT‐29 cells with varying concentrations of the materials for 24 h. CCK‐8 assays revealed >85% cell viability at all tested concentrations, indicating minimal cytotoxicity (Figure S17).

To determine the optimal therapeutic dosage for subsequent in vivo applications, a preliminary dose‐escalation screening was conducted in dextran sulfate sodium (DSS)‐induced mice using low (L), medium (M), and high (H) dosages of BSCS@WO_3_@PDA (Figure S18a). Clinical monitoring revealed that the M dosage most effectively mitigated DSS‐induced body weight loss and reduced disease activity index (DAI) scores (Figure S18b,c). Macroscopic and histological evaluations further corroborated this trend, as the M group exhibited optimally restored colon length, alleviated splenomegaly, and the most significant repair of colonic mucosal architecture (Figure S19). Notably, increasing the dosage to the H level did not result in further significant improvement in therapeutic outcomes. Therefore, the M dosage (1 × 10^8^ CFU BS / 40 mg kg^−1^ WO_3_@PDA) was established as the optimal therapeutic dose for all subsequent investigations.

Consequently, the in vivo biosafety profile was further evaluated in healthy C57BL/6 mice via oral gavage of BSCS@WO_3_@PDA at this optimal dosage for 7 days. No significant changes in body weight were observed during treatment (Figure S20). We next performed a comprehensive analysis of hematological parameters in the mice. Complete blood count analysis showed no significant alterations in white blood cell (WBC), neutrophil (NEUT), red blood cell (RBC), hemoglobin (HGB), or platelet counts (Figure S21a). Key serum biochemical markers, including alanine aminotransferase (ALT), aspartate aminotransferase (AST), albumin (ALB), blood urea nitrogen (BUN), and creatinine (CREA), showed no significant differences compared to controls (Figure S21b).

Histopathological analysis of heart, liver, spleen, lung, and kidney tissues via hematoxylin and eosin (H&E) staining revealed preserved tissue architecture without pathological alterations (Figure S22). To address potential concerns regarding gastrointestinal side effects, H&E‐stained sections of the entire digestive tract (stomach, duodenum, jejunum, ileum, and colon) were examined, revealing no mucosal damage or structural abnormalities (Figure S23). Furthermore, considering the potential immunogenicity of tungsten‐based nanomaterials, we evaluated the inflammatory response in healthy intestinal tissues. Immunofluorescence staining of a macrophage marker (F4/80) together with a pan‐monocyte/macrophage‐associated marker (CD68) showed no significant increase in positive areas compared to the control group (Figure S24). Additionally, immunohistochemical (IHC) analysis confirmed that the expression of pro‐inflammatory cytokines (TNF‐α, IL‐1β, IL‐6) and the anti‐inflammatory cytokine (IL‐10) in healthy mice remained comparable to the control group following BSCS@WO_3_@PDA administration (Figure S25). Collectively, these findings demonstrate the favorable biocompatibility of BSCS@WO_3_@PDA in vitro and in vivo, confirming that the system does not induce gastrointestinal irritation or inflammatory responses, thereby supporting its advancement to therapeutic efficacy investigations.

**FIGURE 4:**
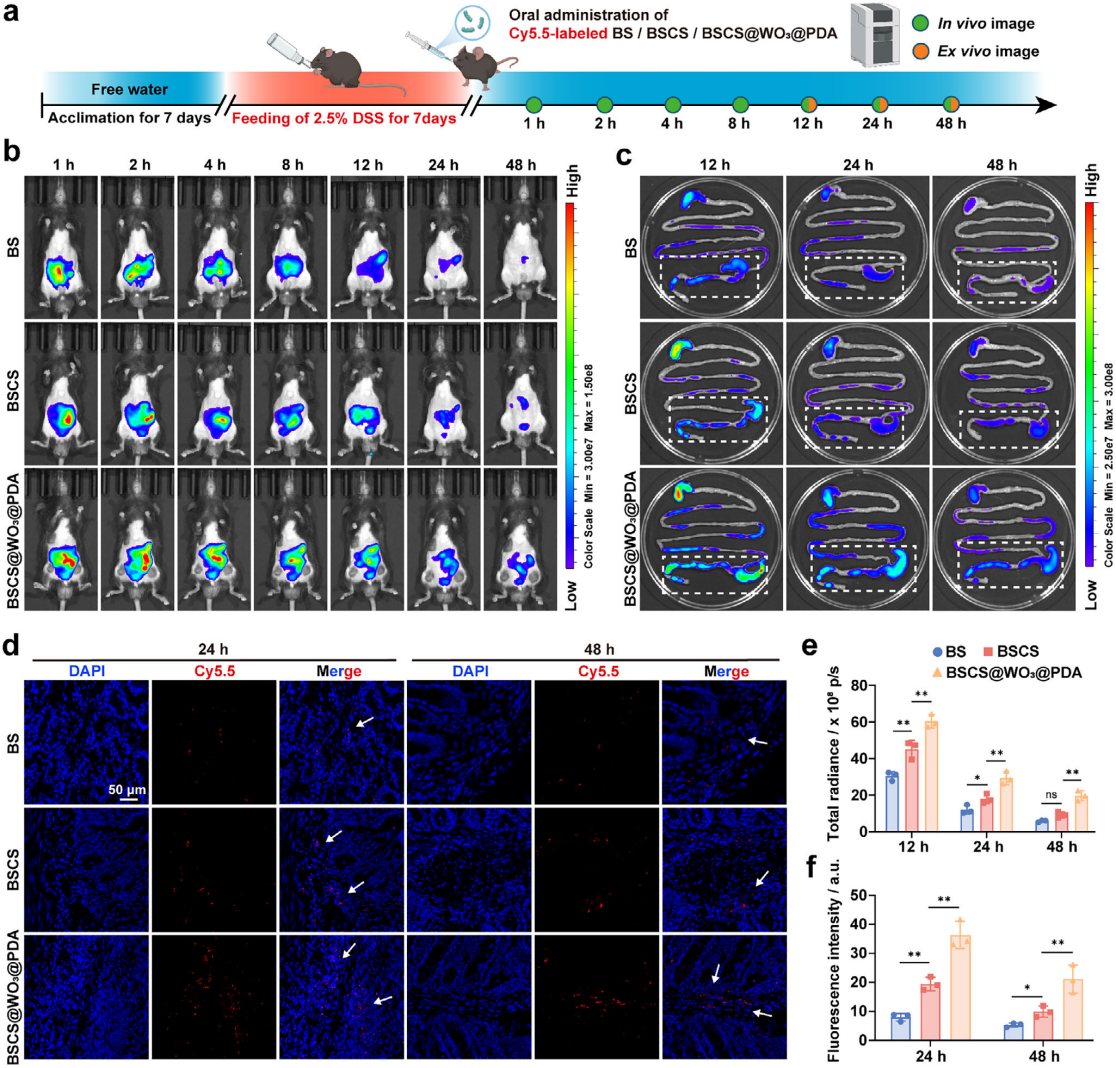
Enhanced intestinal retention of BSCS@WO_3_@PDA in DSS‐induced colitis mice. (a) Schematic diagram of the in vivo imaging experiment. DSS‐induced colitis mice were orally administered Cy5.5‐labeled BS, BSCS, or BSCS@WO_3_@PDA on day 7 for biodistribution monitoring. (b) In vivo fluorescence images of Cy5.5‐labeled probiotics after oral gavage at the indicated time points. (c) Ex vivo fluorescence images of gastrointestinal tracts at 12, 24, and 48 h. (d) CLSM images of colon frozen sections (24 and 48 h): DAPI (blue), Cy5.5‐labeled probiotics (red). Scale bar: 100 µm. (e) Quantitative analysis of fluorescence intensity in the ileocecal and colonic regions (ROIs from panel c). (f) Quantitative analysis of BS signals in colon sections (derived from panel d). Data are presented as mean ± SD (*n* = 3). *p < 0.05, **p < 0.01; ns, not significant.

Prolonged retention of probiotics in the inflamed colon is critical for effective oral therapy. To evaluate the biodistribution and retention efficacy of BSCS@WO_3_@PDA, we used a DSS‐induced colitis mouse model, which is one of the most extensively utilized models for studying IBD [42]. As shown in Figure 4a, following the induction of colitis with 2.5% (w/v) DSS for 7 days, mice were orally administered Cy5.5‐labeled BS, BSCS, or BSCS@WO_3_@PDA, and fluorescence distribution was monitored over 48 h using an in vivo imaging system (IVIS). Throughout the 48 h period, BSCS@WO_3_@PDA sustained a significantly stronger fluorescence intensity than unmodified BS (Figure 4b), indicating enhanced probiotic retention.

Furthermore, intestines and major organs were harvested at 12, 24, and 48 h post‐administration. *Ex vivo* imaging revealed that the BSCS@WO_3_@PDA group exhibited significantly enhanced fluorescence signals specifically localized in the colon, with approximately threefold higher intensity compared to the unmodified BS group (Figure 4c,e). This enhanced accumulation in the colon can be attributed to the abundant catechol groups in the WO_3_@PDA NPs. Under the high‐ROS conditions at the inflammatory site, these groups are oxidized to reactive quinones, which covalently bind to nucleophilic groups in colonic mucins via Michael addition or Schiff base reactions, which may contribute to prolonged adhesion at the diseased site [43]. Furthermore, undetectable fluorescence signals were observed in major organs (heart, liver, spleen, lungs, and kidneys) throughout the monitoring period, confirming minimal systemic dissemination risk following BSCS@WO_3_@PDA administration (Figure S26).

*Ex vivo* analysis of intestinal frozen sections revealed dense fluorescence signals localized within the intestinal epithelium of BSCS@WO_3_@PDA‐treated mice (Figure 4d,f), demonstrating enhanced bacterial retention capacity. Based on these findings, fecal samples were collected 24 h post‐gavage for bacterial culture. Results showed an increase in the total culturable bacterial count within the intestines of mice receiving BSCS@WO_3_@PDA, with more *Bacillus*‐like colonies observed in the cultured flora (Figure S27). Collectively, these results demonstrate that BSCS@WO_3_@PDA achieves adhesion and sustained retention within inflamed colonic tissue, establishing a critical foundation for enhanced probiotic therapeutic efficacy.

**FIGURE 5:**
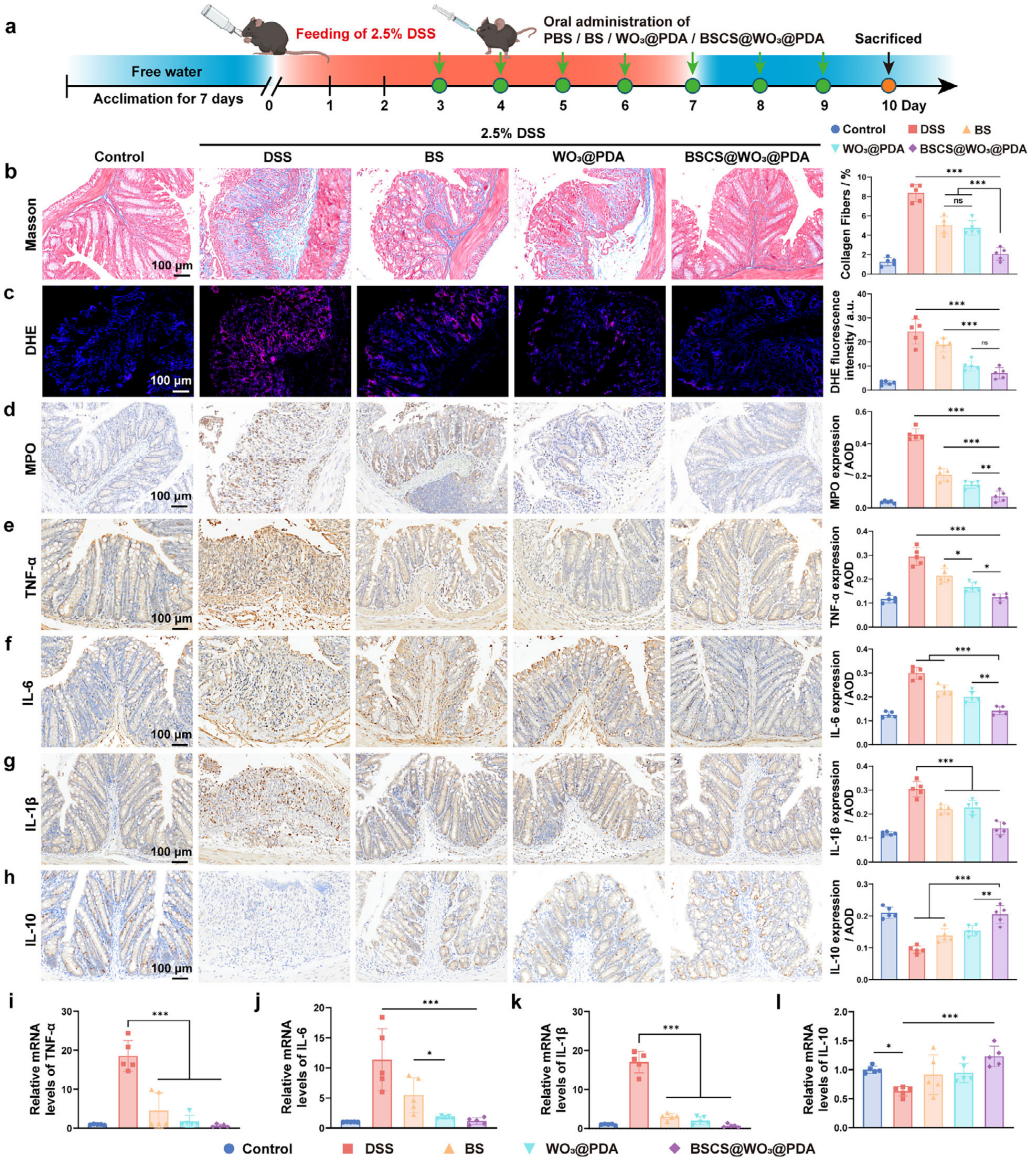
BSCS@WO_3_@PDA suppresses intestinal inflammation in DSS‐induced colitis mice. (a) Schematic diagram of the in vivo therapeutic experiment. C57BL/6 mice were administered 2.5% (w/v) DSS from day 0 to 7 to induce colitis and received daily oral gavage of PBS, BS, WO_3_@PDA NPs, or BSCS@WO_3_@PDA from day 3 to 9. All mice were sacrificed on day 10. Representative images and semi‐quantitative analysis of colon tissues: (b) Masson's staining (blue: collagen fibers). Scale bar: 100 µm; (c) DHE staining. Scale bar: 100 µm; (d) IHC staining of MPO. Scale bar: 100 µm; (e–h) IHC staining for the expression of inflammatory cytokine proteins (TNF‐α, IL‐6, IL‐1β, and IL‐10). Scale bar: 50 µm. (i–l) Relative mRNA expression of inflammatory cytokines (TNF‐α, IL‐6, IL‐1β, and IL‐10) in colon tissues (normalized to β‐actin). Data are presented as mean ± SD (*n* = 5). *p < 0.05, **p < 0.01, ***p < 0.001; ns, not significant.

Leveraging the gastrointestinal stability and exceptional ROS scavenging capacity of BSCS@WO_3_@PDA, we established a 2.5% (w/v) DSS‐induced colitis mouse model to evaluate therapeutic efficacy. The experimental timeline is detailed in Figure 5a. Mice were randomly divided into five groups: control group, DSS group, and three treatment groups receiving BS, WO_3_@PDA NPs, or BSCS@WO_3_@PDA. Therapeutic interventions were administered via oral gavage from day 3 to day 9 post‐induction, with daily doses of 1 × 10^8^ CFU BS and 40 mg kg^−1^ WO_3_@PDA NPs. On day 10, mice were sacrificed, and colons and major organs were collected for evaluation of therapeutic effects.

We first evaluated the anti‐inflammatory effects of BSCS@WO_3_@PDA. Masson's staining of colon tissues revealed substantial collagen deposition in the submucosa and muscular layers of the DSS group, indicating severe intestinal fibrotic damage. In contrast, BSCS@WO_3_@PDA treatment restored collagen deposition to near‐normal levels (Figure 5b), demonstrating the ability of the composite system to ameliorate colitis‐associated tissue fibrosis. This anti‐fibrotic effect is likely attributable to its potent antioxidative capacity [44]. Dihydroethidium (DHE) staining confirmed that colonic epithelial ROS levels increased approximately 8‐fold in the DSS group, whereas BSCS@WO_3_@PDA treatment reduced ROS fluorescence intensity by 60%–80% (Figure 5c). In contrast, BS treatment alone afforded only modest protection, highlighting the essential role of WO_3_@PDA NPs in mediating ROS clearance. Given that oxidative stress is a key driver of inflammatory cell infiltration, we further assessed neutrophil recruitment. IHC staining for myeloperoxidase (MPO), a marker for neutrophils, revealed that BSCS@WO_3_@PDA treatment reduced MPO‐positive cell infiltration by approximately 80% (Figure 5d), indicating its efficacy in suppressing acute inflammation. At the immunoregulatory level, substantial infiltration of F4/80‐positive macrophages and CD68‐positive mononuclear phagocytes was observed in DSS‐induced colonic lesions, which was significantly attenuated by BSCS@WO_3_@PDA treatment (Figure S28). Consistent with these findings, both IHC (Figure 5e–h) and RT‐qPCR analyses (Figure 5i–l) showed that the BSCS@WO_3_@PDA treatment significantly downregulated the expression of pro‐inflammatory cytokines (TNF‐α, IL‐1β, and IL‐6) while upregulating the anti‐inflammatory cytokine IL‐10. These findings suggest that BSCS@WO_3_@PDA not only suppresses inflammation but also actively modulates immune homeostasis, promoting a shift from a pro‐inflammatory state to an anti‐inflammatory and reparative milieu in the colonic mucosa.

### 2.3 Integrated Microbiome Regulation and Metabolome Analysis by Oral Administration of BSCS@WO_3_@PDA

**FIGURE 6:**
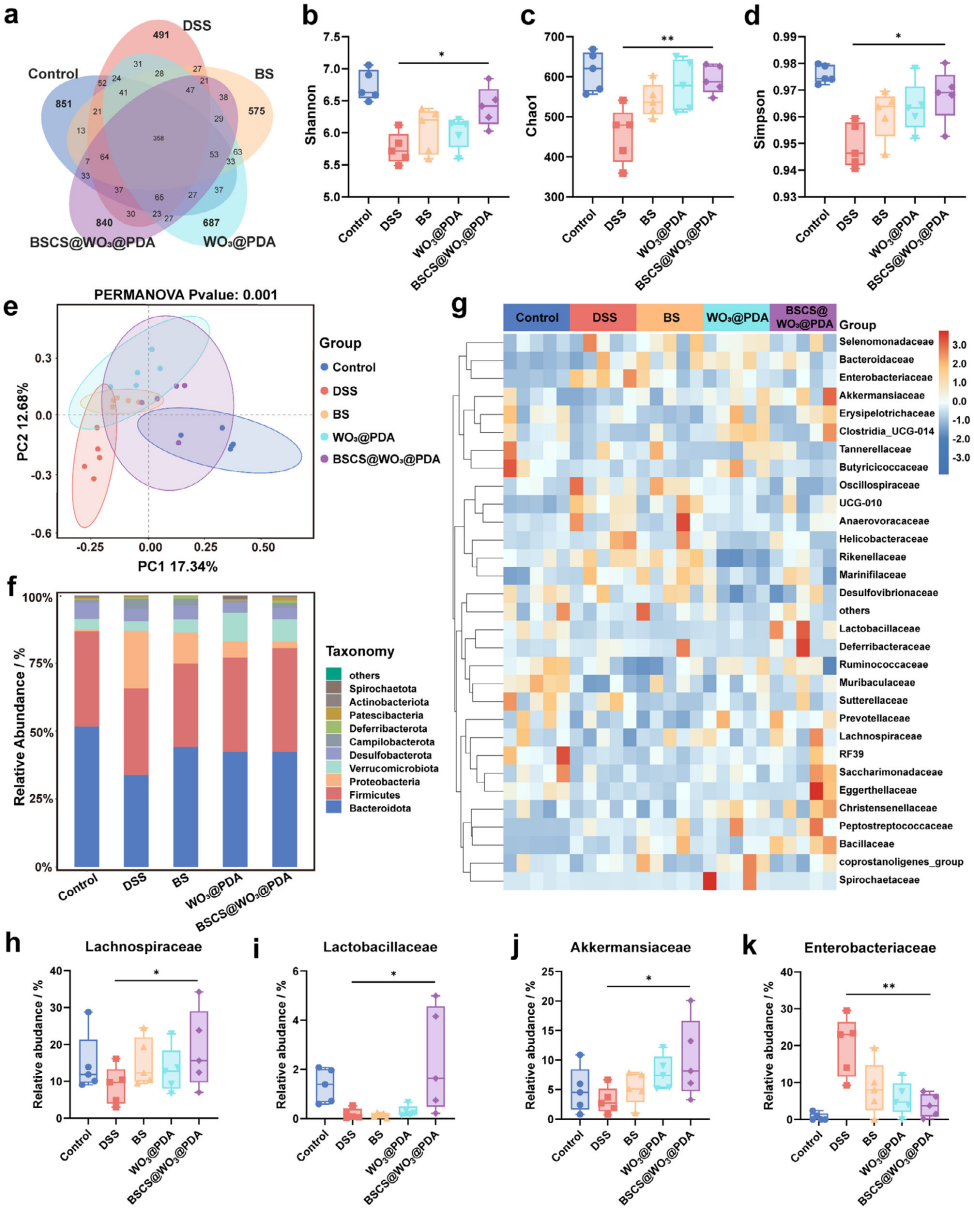
BSCS@WO_3_@PDA restores gut dysbiosis in DSS‐induced colitis mice. Gut microbiota in colitis mice after BSCS@WO_3_@PDA treatment was examined by 16S rRNA sequencing. The fecal samples were collected on day 10. (a) Venn diagram of OTUs. Microbial α‐diversity indices: (b) Shannon index, (c) Chao1 index, (d) Simpson index. (e) PCoA plot based on Bray–Curtis distances. (f) Phylum‐level relative abundance profiles. (g) Family‐level clustered heatmap. Relative abundance at the family level of (h) Lachnospiraceae, (i) Lactobacillaceae, (j) Akkermansiaceae, and (k) Enterobacteriaceae. Data are presented as mean ± SD (*n* = 5). *p < 0.05, **p < 0.01.

Gut dysbiosis, characterized by Enterobacteriaceae over‐proliferation and significant depletion of beneficial microbiota, is closely linked to colitis pathogenesis [4]. Based on this, we performed 16S rRNA gene sequencing on mouse fecal samples. Quality control results are shown in Figure S29. Venn analysis depicted the distribution of unique operational taxonomic units (OTUs) across groups. Notably, the BSCS@WO_3_@PDA group exhibited a significantly higher total OTU count compared to other groups (Figure 6a). Concurrently, α‐diversity indices (Chao1, Shannon, and Simpson) further demonstrated that BSCS@WO_3_@PDA treatment significantly enhanced gut microbial diversity, partially restoring it toward normal levels (Figure 6b–d). Principal coordinates analysis (PCoA) revealed shifts in β‐diversity. Samples from the BSCS@WO_3_@PDA group exhibited high clustering overlap with the control group while remaining distinctly separated from the DSS group (Figure 6e). These results indicate that the treatment effectively modulated the microbial community structure and promoted restoration of microbiota homeostasis, which are key hallmarks of ameliorated gut dysbiosis. Furthermore, alleviation of dysbiosis has been closely linked to therapeutic efficacy in colitis [45].

At the phylum level, consistent with clinical observations in IBD [46], DSS‐induced colitis led to a significant enrichment of the pro‐inflammatory phylum Proteobacteria, while treatment with BSCS@WO_3_@PDA effectively reduced its relative abundance (Figure 6f). Subsequent cluster heatmap analysis at the family level (Figure 6g) revealed that the intervention markedly increased the abundance of beneficial bacteria, including Lactobacillaceae, Lachnospiraceae, and Akkermansiaceae (Figure 6h–j). Lactobacillaceae ferment carbohydrates to produce lactate, which lowers intestinal pH and inhibits pathogen growth [47]. Lachnospiraceae is a major producer of short‐chain fatty acids in the gut, particularly butanoic acid, which serves as a primary energy source for colonic epithelial cells and maintains intestinal epithelial barrier integrity [48]. Notably, Akkermansiaceae is recognized as a next‐generation beneficial microbe due to its potent intestinal barrier‐modulating properties [49]. Importantly, compared to the DSS group, BSCS@WO_3_@PDA treatment significantly suppressed the abundance of pathogenic Enterobacteriaceae (Figure 6k). Although the reduced abundance of beneficial families such as Eggerthellaceae and Muribaculaceae in the DSS group did not achieve statistical significance after intervention, upward trends in their mean abundances were observed (Figure S30).

To further interpret the observed microbiome compositional differences, we performed linear discriminant analysis effect size analysis. In line with the findings above, the DSS group exhibited marked enrichment of IBD‐associated Proteobacteria and pathogenic Enterobacteriaceae (Figures S31 and S32). In contrast, BSCS@WO_3_@PDA treatment effectively suppressed these pathogens while promoting the taxa of beneficial genera such as *Lachnospiraceae_NK4A136_group* and *Lactobacillus*. Collectively, these findings indicate that BSCS@WO_3_@PDA treatment significantly enhances gut microbiota diversity, disrupts the ecological niche occupied by pathogenic bacteria like Enterobacteriaceae during colitis, and supports the enrichment and proliferation of beneficial bacteria, thereby restoring microbiota homeostasis. This restoration is correlated with the amelioration of colitis.

**FIGURE 7:**
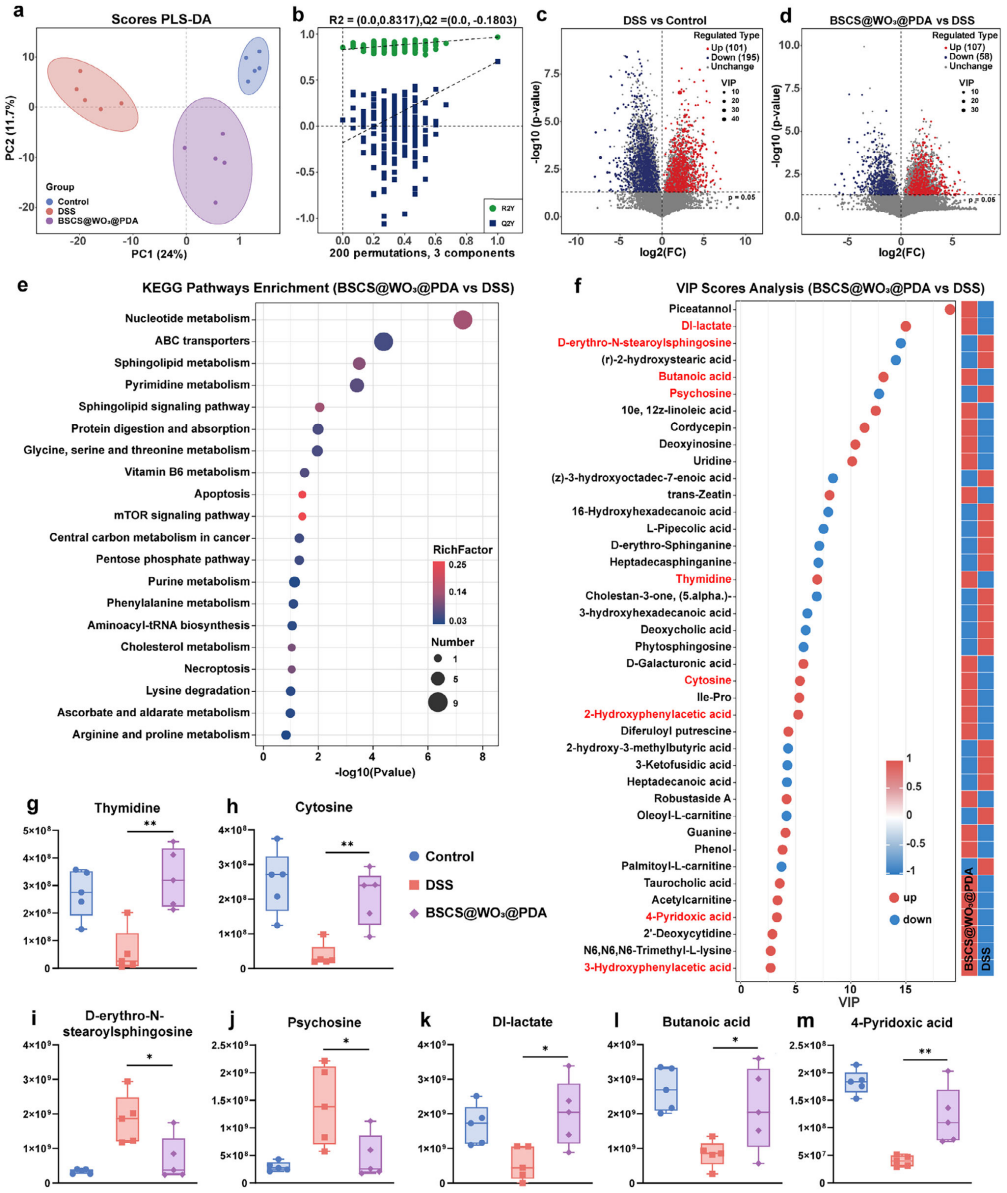
Mechanistic investigation of BSCS@WO_3_@PDA in alleviating DSS‐induced colitis through metabolomic analysis. (a) PLS‐DA score plot of Control, DSS, and BSCS@WO_3_@PDA groups. (b) Permutation test for model validation. Volcano plots of differential metabolites: (c) DSS versus Control groups and (d) BSCS@WO_3_@PDA versus DSS groups. (e) Top 20 enriched KEGG pathways for metabolites differing between BSCS@WO_3_@PDA and DSS groups. (f) VIP scores of the top 40 metabolites differentiating BSCS@WO_3_@PDA from DSS groups. Relative abundance of specific metabolites: (g) thymidine, (h) cytosine, (i) D‐erythro‐N‐stearoylsphingosine, (j) psychosine, (k) DL‐lactate, (l) butanoic acid, and (m) 4‐pyridoxic acid. Data are presented as mean ± SD (*n* = 5). *p < 0.05, **p < 0.01.

To further elucidate the potential mechanisms underlying the therapeutic effect of BSCS@WO_3_@PDA on colitis, we performed untargeted metabolomic profiling on fecal samples from the Control, DSS, and BSCS@WO_3_@PDA‐treated groups. Quality control assessments confirmed the reliability of the metabolomic data (Figure S33). Partial least squares‐discriminant analysis (PLS‐DA) revealed a clear separation of metabolic profiles among the three groups (Figure 7a), and permutation testing confirmed the robustness and reliability of the model (Figure 7b). Volcano plot analysis demonstrated that DSS induction caused significant disturbances in a large number of metabolites, whereas BSCS@WO_3_@PDA treatment substantially reversed these alterations (Figure 7c,d), indicating that the intervention induced systemic metabolic reprogramming.

Kyoto Encyclopedia of Genes and Genomes (KEGG) pathway enrichment analysis showed that the differentially abundant metabolites were primarily enriched in several core functional modules, including nucleotide metabolism, sphingolipid metabolism and signaling pathway, the mTOR signaling pathway, and phenylalanine metabolism, among others (Figure 7e). Analysis based on variable importance in projection (VIP) scores further validated the directional regulation of these pathways (Figure 7f). Specifically, levels of DNA/RNA synthesis precursors such as thymidine and cytosine were significantly restored in the treatment group (Figure 7g,h), providing essential substrates for the proliferation of the damaged intestinal epithelium [50]. Concurrently, elevated levels of arginine (Figure S34a) likely function as signaling molecules to moderately activate the mTOR pathway, thereby potentially supporting anabolic metabolism and facilitating epithelial repair [51]. Meanwhile, the abundance of pro‐apoptotic sphingolipids, such as D‐erythro‐N‐stearoylsphingosine and psychosine, was significantly reduced (Figure 7i,j), suggesting the suppression of sphingolipid‐mediated apoptotic signaling [52, 53]. These metabolic shifts collectively point to a proactive repair phenotype, driven by the synergistic upregulation of cellular biosynthesis and downregulation of apoptotic programs, potentially facilitating transition of the damaged mucosa toward a regenerative state.

This metabolic reprogramming appears to be mediated by a programmed cascade within BSCS@WO_3_@PDA. Specifically, the system mitigates oxidative stress‐induced damage to both intestinal epithelial cells and probiotics while specifically suppressing pathogenic Enterobacteriaceae, thereby reducing inflammatory stimuli and collectively optimizing the gut microenvironment. The observed reduction in Enterobacteriaceae abundance was paralleled by a corresponding decrease in the level of cadaverine (Figure S34b), a key metabolite produced by this bacterial family [54]. This coordinated change is consistent with the intended suppression of pathogenic metabolic activity following BSCS@WO_3_@PDA intervention.

Building upon this optimized microenvironment, BS further enhanced the functionality of beneficial bacteria. Elevated levels of DL‐lactate and butanoic acid were observed (Figure 7k,l), correlating with an increased abundance of Lactobacillaceae and Lachnospiraceae as indicated by 16S rRNA sequencing [47]. Functionally, lactate not only lowers intestinal pH to inhibit pathogens but also serves as a precursor for butanoic acid synthesis. Butanoic acid acts as the primary energy source for colonocytes and plays a central role in maintaining epithelial integrity and exerting anti‐inflammatory effects [55]. Concurrently, the significant recovery in the level of 4‐pyridoxic acid (Figure 7m), a vitamin B6 metabolite, provided essential coenzyme support for protein and amino acid metabolism, thereby promoting intestinal barrier repair and immune regulation [56]. Furthermore, elevated levels of the antioxidant metabolites 3‐hydroxyphenylacetic acid and 2‐hydroxyphenylacetic acid (Figure S34c,d), indicated an enhanced microbial capacity to convert aromatic amino acids into beneficial signaling molecules, reinforcing endogenous antioxidant defense in the host [57]. In summary, this metabolomic study suggests that BSCS@WO_3_@PDA alleviates colitis‐associated metabolic disturbances by reprogramming microbial metabolism to modulate host cell functions, thereby restoring intestinal microecological homeostasis and barrier integrity.

### 2.4 In Vivo Therapeutic Efficacy and Barrier Restoration of BSCS@WO_3_@PDA in Colitis

**FIGURE 8:**
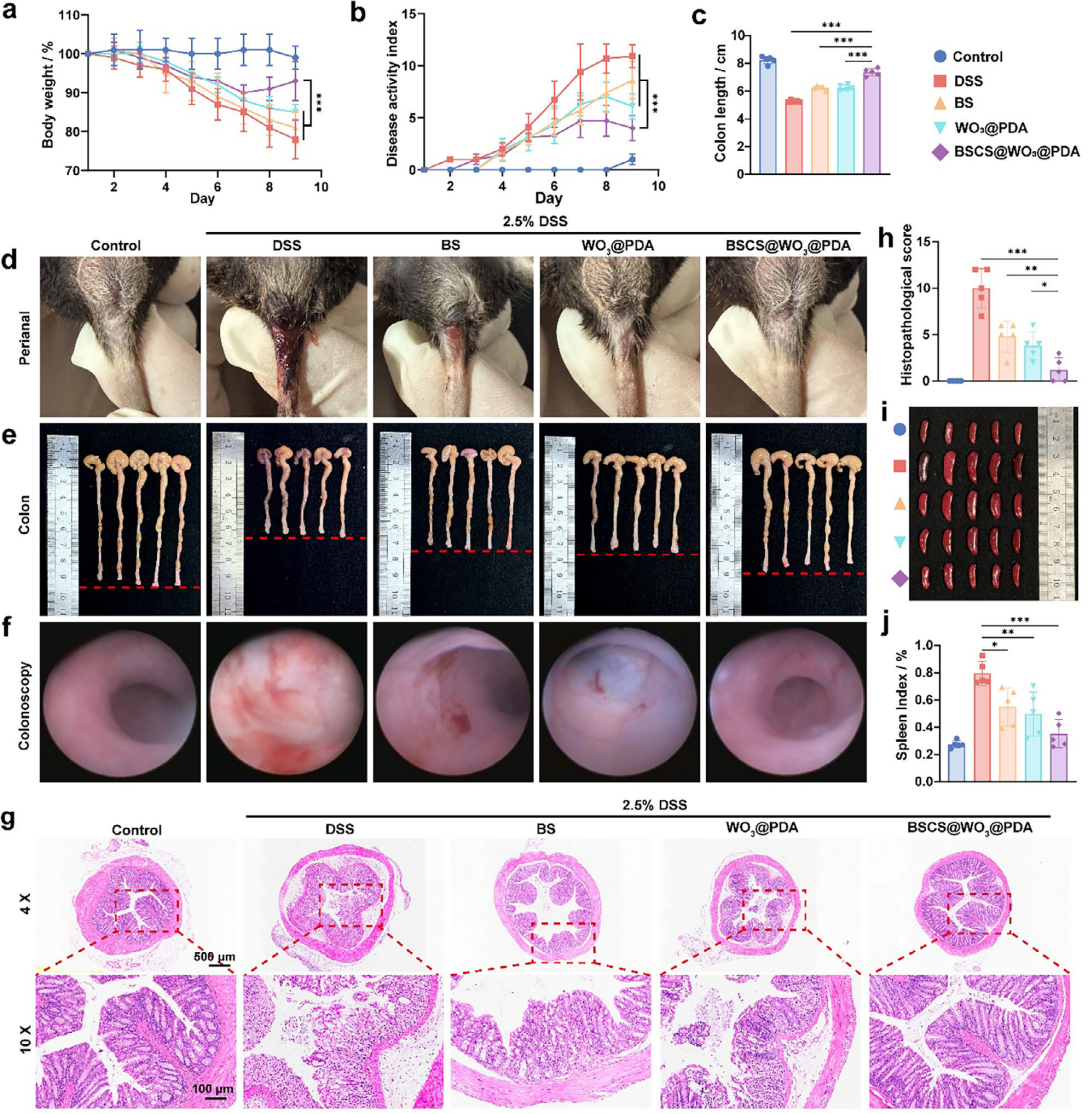
Therapeutic efficacy of BSCS@WO_3_@PDA in DSS‐induced colitis mice. (a) Body weight changes (%) relative to baseline. (b) DAI scores. (c) Quantitative analysis of colon length. Representative photographs of the (d) perianal region and (e) excised colons, and (f) endoscopic image of the colonic mucosa. (g) Representative H&E staining of colon sections. Scale bars: 500 µm (overview), 100 µm (magnified). (h) Histopathological scores of colon tissues. (i) Photographs of excised spleens. (j) Spleen index (spleen weight / body weight × 100%). Data are presented as mean ± SD (*n* = 5). *p < 0.05, **p < 0.01, ***p < 0.001.

Comprehensive evaluation of the therapeutic efficacy of BSCS@WO_3_@PDA in DSS‐induced colitis mice was performed by monitoring disease progression through serial measurements of body weight and the DAI. As a key indicator of colitis severity, body weight changes revealed progressive loss in all DSS‐treated groups starting around day 5 (Figure 8a). In contrast, mice receiving BSCS@WO_3_@PDA showed significant weight recovery, with a final reduction of only approximately 6.7% from baseline. DAI scores further quantified inflammatory severity (detailed criteria in Table S2). The DSS group reached a high DAI score of 10.9 ± 1.1, while BSCS@WO_3_@PDA treatment significantly reduced the score to 4.0 ± 1.2, indicating effective suppression of disease activity (Figure 8b).

Macroscopic examination revealed pronounced perianal bleeding and diarrhea in the DSS group. These symptoms were moderately alleviated in groups treated with BS or WO_3_@PDA NPs alone, whereas mice in the BSCS@WO_3_@PDA group exhibited only minimal signs of bloody stools (Figure 8d). Examination of excised colons revealed significant shortening, wall thickening, and diffuse hyperemia in the DSS group (Figure 8e). Quantitative analysis confirmed an average colon length of 5.26 ± 0.09 cm in the DSS group (Figure 8c). In contrast, BSCS@WO_3_@PDA treatment restored colon length to 7.36 ± 0.27 cm, closely approximating the value observed in the control group (8.24 ± 0.27 cm). Colonoscopic evaluation using the system illustrated in Figure S35 revealed pale, edematous mucosa with diffuse bleeding in the DSS group. In contrast, the BSCS@WO_3_@PDA group exhibited restored pinkish mucosa with only focal congestion (Figure 8f).

Based on these macroscopic findings, we further examined the histopathological changes in colonic ulcerative lesions. H&E staining (Figure 8g) revealed severe colonic damage in the DSS group, characterized by crypt architecture destruction, goblet cell depletion, and extensive inflammatory infiltration. While BS and WO_3_@PDA monotherapies provided partial alleviation, BSCS@WO_3_@PDA treatment preserved near‐normal crypt structures and goblet cell populations. Quantitative histopathological scoring (Figure 8h, criteria in Table S3) confirmed significant attenuation of colonic damage in the BSCS@WO_3_@PDA group. Furthermore, IBD triggers immune system overactivation, leading to compensatory splenomegaly. The spleen index (spleen / body weight ratio) serves as a well‐established metric for assessing systemic inflammation [58]. We observed significant splenomegaly in the DSS group, with the spleen index elevated to 0.79 ± 0.08. Oral administration of BSCS@WO_3_@PDA normalized spleen size, reducing the index to 0.35 ± 0.1 (Figure 8i,j), indicating systemic inflammation mitigation. Collectively, these findings demonstrate that oral BSCS@WO_3_@PDA significantly alleviates inflammatory symptoms in DSS‐induced colitis mice.

**FIGURE 9:**
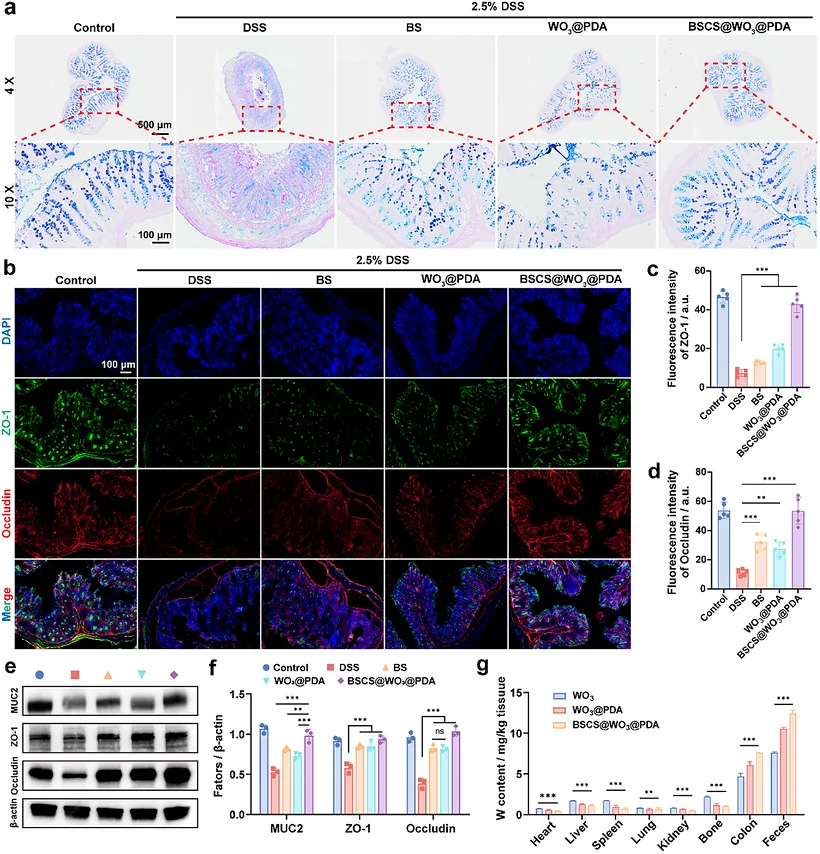
BSCS@WO_3_@PDA restores barrier function. (a) AB‐PAS staining of colon tissues (blue‐purple: mucin). Scale bars: 500 µm (overview), 100 µm (magnified). (b) Immunofluorescence of tight junction proteins: ZO‐1 (green), Occludin (red). Scale bar: 100 µm. Quantitative analysis of (c) ZO‐1 and (d) Occludin fluorescence intensity. (e) Western blot analysis of MUC2, ZO‐1, and Occludin in colon tissues, with β‐actin as the loading control. (f) Corresponding gray analysis of protein levels normalized to β‐actin. (g) Tungsten concentration (mg kg^−1^ tissue) in major organs measured by ICP‐MS after a 7‐day oral administration of WO_3_ NPs, WO_3_@PDA NPs, or BSCS@WO_3_@PDA to DSS‐induced colitis mice. Data are presented as mean ± SD (*n* = 5 in c and d; *n* = 3 in f and g). **p < 0.01, ***p < 0.001; ns, not significant.

Tight junctions, the principal intercellular connections in the intestinal epithelium, are critical for maintaining gut homeostasis by forming a barrier against the translocation of harmful microbes and toxic metabolites into the systemic circulation. Compromise of this barrier exacerbates colitis and may contribute to systemic disorders [59]. Consequently, we evaluated the efficacy of BSCS@WO_3_@PDA in restoring intestinal barrier integrity. Alcian Blue‐Periodic Acid Schiff (AB‐PAS) staining showed that DSS treatment induced a depletion of colonic mucins, impairing barrier function. In contrast, BSCS@WO_3_@PDA treatment preserved goblet cell architecture and significantly increased mucin secretion compared to the DSS group (Figure 9a). Immunofluorescence analysis of tight junction proteins (ZO‐1and Occludin) revealed that BSCS@WO_3_@PDA restored their expression to approximately 90% of control levels, in contrast to the significant reduction seen in the DSS group (Figure 9b–d). Consistent with these findings, western blot analysis confirmed decreased expression of MUC2, ZO‐1, and Occludin in the DSS group, while BSCS@WO_3_@PDA treatment effectively normalized these protein levels (Figure 9e,f, and Figure S36). Furthermore, the restored expression of tight junction proteins and mucin was mechanistically linked to metabolic reprogramming (Figure 7e), where upregulated nucleotide biosynthesis supplied essential precursors for epithelial repair, while downregulated pro‐apoptotic sphingolipid signaling enhanced cell survival. This shift to a pro‐anabolic state drove barrier restoration beyond the passive recovery achieved through anti‐inflammatory measures alone.

The in vivo biodistribution of tungsten was evaluated in DSS‐induced colitis mice following daily oral administration of WO_3_ NPs, WO_3_@PDA NPs, or BSCS@WO_3_@PDA (WO_3_ NPs dose: 40 mg kg^−1^) for 7 days. ICP‐MS analysis of digested major organs showed significantly lower tungsten accumulation in the heart, liver, spleen, lungs, kidneys, and bones of mice treated with BSCS@WO_3_@PDA compared to those receiving WO_3_ NPs. In contrast, tungsten levels in the colon and feces were approximately 2‐fold higher in the BSCS@WO_3_@PDA group (Figure 9g). Unlike soluble tungstate ions, WO_3_ NPs inherently reduce systemic distribution risks through enhanced colonic mucus adhesion [26]. However, premature release of tungsten ions under gastric acidic conditions remains a potential concern. The acid‐resistant PDA coating effectively prevents ion leakage prior to reaching the colon, while the elevated ROS levels at inflamed sites trigger controlled degradation of PDA and subsequent localized tungsten ions release. This responsive release mechanism minimizes off‐target accumulation. Additionally, BS further enhances the retention of WO_3_@PDA NPs, thereby increasing colonic tungsten enrichment.

In addition, hematological analysis revealed that BSCS@WO_3_@PDA treatment restored the DSS‐induced elevations in peripheral WBC and NEUT counts, as well as reductions in RBC and HGB levels, to near‐normal values, indicating mitigation of systemic inflammation and anemia (Figure S37a). Consistent with these findings, serum biochemical analysis further showed that BSCS@WO_3_@PDA significantly reduced the abnormally elevated levels of ALT, AST, BUN, and CREA while increasing ALB concentration relative to the DSS group, suggesting protection against liver and kidney injury (Figure S37b). H&E staining of major organs (heart, liver, spleen, lung, and kidney) revealed mild liver injury in the DSS group, characterized by hepatocyte swelling and cytoplasmic vacuolization, whereas BSCS@WO_3_@PDA treatment markedly alleviated these pathological changes. No significant pathological changes were observed in other organs across all groups (Figure S38). Overall, these findings demonstrate that BSCS@WO_3_@PDA does not induce systemic toxicity and may attenuate extra‐intestinal complications like colitis‐associated liver injury.

Collectively, the above results suggest that BSCS@WO_3_@PDA effectively alleviates symptoms of DSS‐induced colitis, maintains the integrity of the tight junction barrier, and is associated with no significant adverse effects while reducing the off‐target distribution of tungsten.

## 3 Conclusions

In this study, we developed a novel programmed cascade metabolic intervention for IBD using a multifunctional composite probiotic system, BSCS@WO_3_@PDA, designed to disrupt the vicious cycle between oxidative stress and gut microbiota dysbiosis, thereby restoring intestinal microenvironment homeostasis. Overcoming the spatiotemporal mismatch typically associated with simple physical mixtures, this rationally integrated system maintains gastrointestinal stability during oral delivery, enabling effective adhesion to the inflamed colon. Specifically, the PDA coating exerts a precise dual‐responsive function: it ensures structural integrity in acidic environments to prevent premature leakage of tungsten ions, while undergoing responsive degradation in high‐ROS inflammatory microenvironments to achieve controlled tungsten release alongside efficient ROS clearance. The locally released tungsten ions selectively inhibit the anaerobic respiration of Enterobacteriaceae by suppressing molybdenum‐dependent nitrate reductase. Consequently, working in synergy with the protected BS, this cascade intervention promotes the enrichment of beneficial microbes, leading to increased levels of metabolites such as lactate and butanoic acid. These metabolic changes may improve energy metabolism in intestinal epithelial cells. Untargeted metabolomics further revealed that this therapeutic strategy upregulates nucleotide biosynthesis pathways and downregulates pro‐apoptotic sphingolipid signaling, thereby being associated with microbiota‐host metabolic reprogramming, which can promote mucosal healing. Collectively, this integrated approach simultaneously targeting oxidative stress, pathogenic bacteria, and microbiota remodeling offers a promising strategy for the treatment of IBD and other microecology‐related inflammatory diseases.

## 4 Experimental Section

### 4.1 Preparation of WO_3_@PDA NPs

WO_3_@PDA NPs were synthesized via the self‐polymerization of dopamine. Briefly, WO_3_ NPs (1 mg mL^−1^) were dispersed in 10 mM Tris buffer (pH 8.5) by sonication for 30 min. Subsequently, dopamine hydrochloride was introduced into the suspension at a final concentration of 1 mg mL^−1^. The mixture was stirred at 1200 rpm for 2 h at room temperature, during which the color changed from milky white to black, indicating the successful formation of the PDA shell. The resulting product was collected by centrifugation (11 000 rpm, 10 min), washed twice with absolute ethanol, and redispersed in absolute ethanol for storage at 4°C.

### 4.2 Preparation of BSCS@WO_3_@PDA

BS was cultured in LB‐Miller medium (consisting of 8 g L^−1^ tryptone, 2 g L^−1^ yeast extract, and 10 g L^−1^ NaCl) at 37°C with shaking at 200 rpm until the exponential growth phase. Bacterial cells were harvested from a culture containing approximately 1 × 10^9^ CFU by centrifugation (6000 rpm, 5 min). The pellet was washed twice with PBS and then resuspended in 5 mL of a 2 mg mL^−1^ CS solution (pH 6.0). The mixture was stirred at 1200 rpm for 30 min to facilitate coating, forming the BSCS complex. The BSCS was collected by centrifugation (6000 rpm, 5 min) and washed twice with PBS to remove unbound CS. Simultaneously, 10 mg of WO_3_@PDA NPs were dispersed in 5 mL of PBS buffer (pH 6.0) via sonication, and the prepared BSCS was added to this suspension. The combined mixture was stirred at 1200 rpm at room temperature for 2 h. Finally, the product was collected by centrifugation (6000 rpm, 5 min), yielding the BSCS@WO_3_@PDA complex, which was stored in PBS at 4°C for subsequent use.

### 4.3 Colocalization Study of BSCS@WO_3_@PDA

FITC‐labeled BS was incubated with FITC (10 µg mL^−1^) in PBS at 37°C, shaking at 200 rpm for 30 min, and then washed twice with PBS. Rhodamine B‐labeled WO_3_@PDA NPs were synthesized by adding Rhodamine B (50 µg mL^−1^) to the reaction mixture during the PDA polymerization step. Using these pre‐labeled components, the BSCS@WO_3_@PDA complex was prepared as described in the aforementioned procedure. Subsequently, a sample of the composite suspension was placed on a glass slide and imaged via CLSM to verify colocalization (FITC, *Excitation*: 494 nm, *Emission*: 519 nm; Rhodamine B, *Excitation*: 554 nm, *Emission*: 576 nm).

### 4.4 Tungsten Release Experiment

WO_3_@PDA NPs were incubated in SGF (pH 1.5), SIF (pH 6.8), or SCF (pH 7.4) at 37°C with shaking at 100 rpm to mimic gastrointestinal transit. Samples were collected at 2 h (in SGF), 4 and 6 h (in SIF), and 12 and 24 h (in SCF), followed by centrifugation (11 000 rpm, 10 min). The supernatant was then collected, diluted with ultrapure water, and filtered through a 0.45 µm membrane. The tungsten ion concentration in the filtrate was quantified using ICP‐MS to plot the release profiles.

### 4.5 Evaluation of Gastrointestinal Resistance in BSCS@WO_3_@PDA

Suspensions of BS, BSCS, or BSCS@WO_3_@PDA (bacterial concentration: 1 × 10^9^ CFU mL^−1^) were incubated with SGF, SIF, or PBS containing 1.0 mM H_2_O_2_ at 37°C with agitation at 200 rpm. At designated time points (SGF:1 and 2 h; SIF: 2 and 4 h; H_2_O_2_: 2 and 4 h), samples were centrifuged (6000 rpm, 5 min) and resuspended in PBS. After serial dilution, the suspensions were spread onto LB agar plates and incubated overnight at 37°C for colony counting. In parallel, 50 µL of the PBS‐resuspended bacterial solution was inoculated into fresh LB broth. The culture was incubated at 37°C with shaking at 200 rpm. The OD_600_ was monitored hourly for 12 h, and growth curves were plotted accordingly.

### 4.6 Evaluation of ROS Scavenging Capacity

The ROS scavenging capacities of WO_3_@PDA NPs and BSCS@WO_3_@PDA were evaluated using four established methods. Sample suspensions (0–200 µg mL^−1^) were prepared, with BSCS@WO_3_@PDA tested at equivalent WO_3_@PDA concentrations to assess the influence of bacterial integration. Scavenging efficiencies were quantified via UV–vis spectrophotometry: 1. DPPH• assay: Samples were mixed with DPPH• solution (0.1 mM), incubated in the dark for 10 min, and measured at 517 nm. 2. •OH assay: Samples were added to a Fenton reaction system (0.36 mM FeSO_4_, 0.2 mM H_2_O_2_) containing 0.5 mM ABTS. After 5 min, the absorbance of oxidized ABTS was recorded at 756 nm. 3. •O_2_^−^ assay: •O_2_^−^ was generated via a xanthine/xanthine oxidase system (per SOD kit instructions). Following a 40 min incubation at 37°C, absorbance was measured at 550 nm. 4. H_2_O_2_ assay: The degradation of indigo carmine (IC, 75 µM) by H_2_O_2_ (5 mM) was monitored after 24 h at 37°C by measuring the residual absorbance at 610 nm.

### 4.7 Cellular ROS‐Scavenging Activity Effect of WO_3_@PDA NPs

HT‐29 cells were seeded in 6‐well plates (5 × 10^5^ cells well^−1^) and cultured for 24 h. Serum‐free McCoy's 5A medium containing 100 ng mL^−1^ LPS and WO_3_@PDA NPs (20, 40, or 80 µg mL^−1^) was prepared. After removing the original medium and washing cells twice with PBS, cells were incubated with the LPS/WO_3_@PDA NPs‐containing medium for 12 h at 37°C. Subsequently, cells were washed with PBS and incubated with medium containing 10 µM DCFH‐DA for 30 min. Fluorescence images were captured using a fluorescence microscope. For flow cytometry analysis, the stained cells were washed twice with PBS, detached with 0.25% trypsin‐EDTA, and centrifuged (1000 rpm, 5 min). Cell pellets were resuspended in PBS and analyzed immediately by flow cytometry. FITC‐positive cells were quantified, and mean fluorescence intensity was analyzed using FlowJo software.

### 4.8 Inhibitory Effect of WO_3_@PDA NPs on Nitrate Reductase Activity and Anaerobic Growth of *E. Coli*

To induce nitrate reductase expression, overnight cultures of *E. coli* DH5α were diluted 1:100 in fresh LB broth supplemented with 40 mM sodium nitrate (NaNO_3_). WO_3_@PDA NPs (1 mg mL^−1^) were added to the culture medium. The mixtures were transferred to anaerobic jars (with AnaeroPack sachets) and incubated at 37°C for 6 h under oxygen‐free conditions. Dissimilatory nitrate reductase (Nar) activity was quantified using a Nitrate Reductase Activity Assay Kit, measuring the reduction of nitrate to nitrite at 540 nm. For anaerobic growth assessment, identically prepared bacterial cultures were separately incubated at 37°C overnight under anaerobic conditions. Colony enumeration was subsequently performed following standard protocols. Specifically, when evaluating the bacteriostatic effect of the composite BSCS@WO_3_@PDA, MacConkey agar was employed to selectively enumerate *E. coli* colonies, thereby excluding the interference of BS.

### 4.9 Establishment of DSS‐Induced Colitis Mice

Male C57BL/6 mice (6–8 weeks old, body weight 22–25 g) were acclimatized for 7 days prior to experimentation. Colitis was induced by administration of 2.5% (w/v) DSS dissolved in sterile drinking water for 7 consecutive days. Disease progression was monitored daily through the DAI, quantifying weight loss, stool consistency, and fecal bleeding. Detailed DAI scoring criteria are provided in Table S2. The experimental procedures involving animals were approved by the Animal Ethics Committee of Chongqing University (Approval No. CQU‐IACUC‐RE‐202205‐001) and were performed in accordance with institutional guidelines.

### 4.10 In Vivo Fluorescence Imaging

Male C57BL/6 mice (*n* = 3 per group) received 2.5% (w/v) DSS in sterile drinking water for 7 days to induce colitis. Prior to imaging, abdominal hair was removed. Mice were then orally gavaged 0.1 mL of either Cy5.5‐labeled BS, Cy5.5‐labeled BSCS, or Cy5.5‐labeled BSCS@WO_3_@PDA. Whole‐body fluorescence imaging was performed at 1, 2, 4, 8, 12, 24, and 48 h post‐administration using an IVIS Spectrum in vivo imaging system. At 12, 24, and 48 h, subgroups of mice were euthanized. The gastrointestinal tract, heart, liver, spleen, lungs, and kidneys were harvested for *ex vivo* fluorescence imaging. Additionally, colon tissues were rapidly embedded in OCT compound, frozen, sectioned, counterstained with DAPI, and visualized by CLSM (Cy5.5, *Excitation*: 670 nm, *Emission*: 710 nm).

### 4.11 Therapeutic Efficacy of BSCS@WO_3_@PDA in DSS‐Induced Colitis Mice

Male C57BL/6 mice (6–8 weeks old, body weight 22–25 g) were randomly divided using the random number table method into 5 groups (*n* = 5 per group): 1. Control; 2. DSS; 3. DSS + BS (1 × 10^8^ CFU); 4. DSS + WO_3_@PDA (40 mg kg^−1^); and 5. DSS + BSCS@WO_3_@PDA (1 × 10^8^ Tungsten ions BS / 40 mg kg^−1^ WO_3_@PDA). After 7 days of acclimatization, colitis was induced by administering 2.5% (w/v) DSS in drinking water from day 1 to 7, followed by regular sterile water. From day 3 to 9, mice received a daily oral gavage (0.1 mL) of their respective formulations. DAI was recorded daily. On day 10, subgroups of mice were humanely euthanized via cervical dislocation under deep isoflurane anesthesia. Colons were excised, measured for length, and processed as follows: 1) Fixed in 4% paraformaldehyde for paraffin embedding and histology analysis; 2) Fixed in Carnoy's fixative for AB‐PAS staining; 3) Embedded in OCT compound and flash‐frozen for cryosectioning; and 4) Snap‐frozen in liquid nitrogen and stored at ‐80°C for subsequent protein/RNA extraction. Fecal samples were collected for 16S rRNA sequencing of the gut microbiota and non‐targeted metabolomics analysis.

### 4.12 Colonoscopy

Following completion of the therapeutic regimen, mice were fasted for 12 h to clear the feces. Under anesthesia maintained by continuous inhalation of 2% isoflurane via a nose cone, in vivo colonoscopy was performed. A flexible micro‐endoscope (diameter: 0.9 mm) was gently inserted into the colon via the rectum under real‐time visualization. Physiological saline (0.9% NaCl) was continuously infused to maintain luminal distension during the procedure. Images of the colonic mucosa were captured to document the morphological features.

### 4.13 In Vivo Biodistribution of Tungsten

To assess the in vivo biodistribution of tungsten, DSS‐induced colitis mice were orally administered WO_3_ NPs, WO_3_@PDA NPs, or BSCS@WO_3_@PDA (WO_3_ dose: 40 mg kg^−1^) for 7 days. After euthanasia, primary tissues from major organs (heart, liver, spleen, lungs, kidneys, and colon), bone, and feces were collected and weighed. Samples underwent acid digestion (HNO_3_:H_2_O_2_ = 4:1 v/v) at 100°C for 1 h. Digested solutions were diluted with ultrapure water, filtered through 0.45 µm membranes, and subjected to ICP‐MS for tungsten quantification.

### 4.14 Microbiome (16S rRNA) Sequencing and Analysis

Upon completion of the experimental period, fecal samples were collected for 16S rRNA gene sequencing. Genomic DNA was extracted, and the V3–V4 hypervariable regions of the bacterial 16S rRNA gene were amplified with region‐specific primers. Sequencing libraries were prepared and sequenced on the Illumina NovaSeq platform (performed by Applied Protein Technology Co., Ltd., Shanghai, China). Alpha diversity was assessed using indices including observed species, Chao1, Shannon, and Simpson. Beta diversity was evaluated by PCoA based on Bray–Curtis dissimilarity. Taxonomic composition was profiled at both the phylum and family levels.

### 4.15 Untargeted Metabolomics Sequencing and Analysis

Fecal samples were collected from mice in each group and analyzed by LC‐MS/MS (metabolomic sequencing was performed by Applied Protein Technology Co., Ltd., Shanghai, China). Raw MS data were converted to mzXML format using ProteoWizard MSConvert and processed with XCMS software. After quality control, multivariate analyses, including principal component analysis and PLS‐DA, were performed to evaluate metabolic differences between groups. Additionally, KEGG pathway enrichment and significance analyses of metabolites were conducted.

### 4.16 Statistical Analysis

All experiments were conducted with at least three independent replicates. Data are presented as mean ± SD. Quantitative analysis of image data was performed using ImageJ software (version 1.53t). Statistical analyses were performed using GraphPad Prism software (version 10.0). Differences between the two groups were evaluated by unpaired, two‐tailed Student's t‐tests. Comparisons among multiple groups were analyzed by one‐way analysis of variance followed by Tukey's post‐hoc test for multiple comparisons. Statistical significance was defined as *p < 0.05, **p < 0.01, ***p < 0.001; ns, not significant.

## Author Contributions

**Y. Yang**: Conceptualization, Methodology, Investigation, Visualization, Writing – Original Draft, Writing – Review & Editing; **L. Wang**: Conceptualization, Methodology, Investigation, Visualization, Writing – Original Draft, Writing – Review & Editing; **G. Chen**: Conceptualization, Methodology, Investigation, Writing – Original Draft; **Y. Zhang**: Methodology, Investigation; **J. Shi**: Methodology, Investigation; **W. Fan**: Methodology, Investigation; **M. Yu**: Conceptualization, Supervision, Writing – Review & Editing; **J. Zhang**: Conceptualization, Supervision, Funding acquisition, Resources, Writing – Review & Editing; **H. Yang**: Conceptualization, Supervision, Funding acquisition, Resources, Writing – Review & Editing.

## Funding

This work was supported by the National Key R&D Program of China (Grant No. 2022YFA1304000), the National Natural Science Foundation of China (NSFC, Grant No. 82300634, 22475028), and Chongqing Medical Scientific Research Project (joint project of Chongqing Health Commission and Science and Technology Bureau) (Grant No. 2025QNXM033 and 2025MSXM115).

## Conflicts of Interest

The authors declare no conflicts of interest.

## Supporting information




**Supporting File**: advs76291‐sup‐0001‐SuppMat.docx.

## Data Availability

The data that support the findings of this study are available from the corresponding author upon reasonable request.

## References

[1] G. Rogler , A. Singh , A. Kavanaugh , and D. T. Rubin , “Extraintestinal Manifestations of Inflammatory Bowel Disease: Current Concepts, Treatment, and Implications for Disease Management,” Gastroenterology 161 (2021): 1118–1132. 10.1053/j.gastro.2021.07.042 PMC8564770 34358489

[2] Y. Zeng , M. Fan , Q. Zhou , et al., “Reactive Oxygen Species‐Activated CO Versatile Nanomedicine With Innate Gut Immune and Microbiome Remodeling Effects for Treating Inflammatory Bowel Disease,” Advanced Functional Materials 33 (2023): 2304381.

[3] S. L. Vogt , A. Serapio‐Palacios , S. E. Woodward , et al., “Enterohemorrhagic Escherichia Coli Responds to Gut Microbiota Metabolites by Altering Metabolism and Activating Stress Responses,” Gut Microbes 15 (2023): 2190303. 10.1080/19490976.2023.2190303 PMC10038027 36951510

[4] S. Kitamoto , C. J. Alteri , M. Rodrigues , et al., “Dietary L‐Serine Confers a Competitive Fitness Advantage to Enterobacteriaceae in the Inflamed Gut,” Nature Microbiology 5 (2020): 116–125. 10.1038/s41564-019-0591-6 PMC6925351 31686025

[5] T. E. Adolph , M. Meyer , J. Schwärzler , L. Mayr , F. Grabherr , and H. Tilg , “The Metabolic Nature of Inflammatory Bowel Diseases,” Nature Reviews Gastroenterology & Hepatology 19 (2022): 753–767. 10.1038/s41575-022-00658-y 35906289

[6] T. L. Parigi , F. D'Amico , M. T. Abreu , et al., “Difficult‐to‐Treat Inflammatory Bowel Disease: Results From an International Consensus Meeting,” The Lancet Gastroenterology & Hepatology 8 (2023): 853–859. 10.1016/S2468-1253(23)00154-1 37423233

[7] A. S. Faye , K. H. Allin , A. T. Iversen , et al., “Antibiotic Use as a Risk Factor for Inflammatory Bowel Disease Across the Ages: A Population‐Based Cohort Study,” Gut 72 (2023): 663–670. 10.1136/gutjnl-2022-327845 PMC9998355 36623926

[8] M. Fassarella , E. E. Blaak , J. Penders , A. Nauta , H. Smidt , and E. G. Zoetendal , “Gut Microbiome Stability and Resilience: Elucidating the Response to Perturbations in Order to Modulate Gut Health,” Gut 70 (2021): 595–605. 10.1136/gutjnl-2020-321747 33051190

[9] S. Asgari , A. Pourjavadi , T. R. Licht , et al., “Polymeric Carriers for Enhanced Delivery of Probiotics,” Advanced Drug Delivery Reviews 1 (2020): 161–162. 10.1016/j.addr.2020.07.014 32702378

[10] M. T. Khan , C. Dwibedi , D. Sundh , et al., “Synergy and Oxygen Adaptation for Development of Next‐Generation Probiotics,” Nature 620 (2023): 381–385. 10.1038/s41586-023-06378-w PMC10412450 37532933

[11] P. Piewngam , Y. Zheng , T. H. Nguyen , et al., “Pathogen Elimination by Probiotic *Bacillus* via Signalling Interference,” Nature 562 (2018): 532–537. 10.1038/s41586-018-0616-y PMC6202238 30305736

[12] J. Yang , S. Tan , S. Ge , et al., “Cyanobacteria–Probiotics Symbionts for Modulation of Intestinal Inflammation and Microbiome Dysregulation in Colitis,” Proceedings of the National Academy of Sciences 121 (2024): 2403417121. 10.1073/pnas.2403417121 PMC11670216 39680761

[13] Y. Zhu , Z. Fang , J. Bai , et al., “Orally Administered Functional Polyphenol‐Nanozyme‐Armored Probiotics for Enhanced Amelioration of Intestinal Inflammation and Microbiota Dysbiosis,” Advanced Science 12 (2025): 2411939. 10.1002/advs.202411939 PMC12061243 40067175

[14] J. Yang , G. Zhang , M. Peng , et al., “Bionic Regulators Break the Ecological Niche of Pathogenic Bacteria for Modulating Dysregulated Microbiome in Colitis,” Advanced Materials 34 (2022): 2204650. 10.1002/adma.202204650 35924734

[15] J. Zhou , M. Li , Q. Chen , et al., “Programmable Probiotics Modulate Inflammation and Gut Microbiota for Inflammatory Bowel Disease Treatment After Effective Oral Delivery,” Nature Communications 13 (2022): 3432. 10.1038/s41467-022-31171-0 PMC9198027 35701435

[16] B. Deng , S. Lin , Y. Wang , et al., “Hyaluronic Acid‐Nanocoated Bacteria Generate an Anti‐Inflammatory Tissue‐Repair Effect in Impaired Gut and Extraintestinal Organs,” Advanced Materials 37 (2025): 2412783. 10.1002/adma.202412783 39568244

[17] P. Peng , T. Feng , X. Yang , et al., “Gastrointestinal Microenvironment Responsive Nanoencapsulation of Probiotics and Drugs for Synergistic Therapy of Intestinal Diseases,” ACS Nano 17 (2023): 14718–14730. 10.1021/acsnano.3c02646 37490035

[18] Y. Yin , Z. Li , H. Gao , et al., “Microfluidics‐Derived Microparticles with Prebiotics and Probiotics for Enhanced in Situ Colonization and Immunoregulation of Colitis,” Nano Letters 24 (2024): 1081–1089. 10.1021/acs.nanolett.3c03580 38227962

[19] L. Zhu , T. Yu , W. Wang , et al., “Responsively Degradable Nanoarmor‐Assisted Super Resistance and Stable Colonization of Probiotics for Enhanced Inflammation‐Targeted Delivery,” Advanced Materials 36 (2024): 2308728. 10.1002/adma.202308728 38241751

[20] P. Peng , T. Feng , X. Yang , et al., “Bioorthogonal Conjugation and Responsive Nanocoating of Probiotics for Inflammatory Bowel Disease,” Journal of Controlled Release 374 (2024): 538–549. 10.1016/j.jconrel.2024.08.036 39186984

[21] P. Peng , R. Ding , J. Wang , et al., “Antioxidant and Immunomodulator Nanoengineered Probiotics for Synergistic Therapy of Inflammatory Bowel Disease,” Chemical Engineering Journal 512 (2025): 162678.

[22] W. Zhu , M. G. Winter , M. X. Byndloss , et al., “Precision Editing of the Gut Microbiota Ameliorates Colitis,” Nature 553 (2018): 208–211. 10.1038/nature25172 PMC5804340 29323293

[23] Y. Qin , Z. Wang , H. Chen , G. Nie , and R. Zhao , “Oral Nanoparticle Therapy for Inflammatory Bowel Disease by Paneth Cell Regulation and Mucus Layer Remodeling,” Matter 8 (2025): 102084.

[24] C. Gu , Z. Wang , Y. Pan , S. Zhu , and Z. Gu , “Tungsten‐Based Nanomaterials in the Biomedical Field: A Bibliometric Analysis of Research Progress and Prospects,” Advanced Materials 35 (2023): 2204397. 10.1002/adma.202204397 35906814

[25] H. Du , X. Yu , T. Yang , L. Lu , J. Sun , and H. Xie , “Unveiling the Dark Side of Tungsten: A Comprehensive Review of Its Toxicity,” Ecotoxicology and Environmental Safety 301 (2025): 118505. 10.1016/j.ecoenv.2025.118505 40499350

[26] Y. Qin , R. Zhao , H. Qin , et al., “Colonic Mucus‐Accumulating Tungsten Oxide Nanoparticles Improve the Colitis Therapy by Targeting Enterobacteriaceae,” Nano Today 39 (2021): 101234.

[27] J. Deng , Y. Hu , P. Zhu , et al., “Probiotic Delivery for Editing of the Gut Microbiota to Mitigate Colitis and Maintain Hepatic Homeostasis Via Gut–Liver Axis,” ACS Nano 19 (2025): 10500–10514. 10.1021/acsnano.5c00325 40047584

[28] F. Ding , X. Gao , X. Huang , et al., “Polydopamine‐Coated Nucleic Acid Nanogel for siRNA‐Mediated Low‐Temperature Photothermal Therapy,” Biomaterials 245 (2020): 119976. 10.1016/j.biomaterials.2020.119976 32213362

[29] Q. Li , W. He , W. Li , et al., “Band‐Aid‐Like Self‐Fixed Barrier Membranes Enable Superior Bone Augmentation,” Advanced Science 10 (2023): 2206981. 10.1002/advs.202206981 PMC10238180 37029705

[30] C. Pan , J. Li , W. Hou , et al., “Polymerization‐Mediated Multifunctionalization of Living Cells for Enhanced Cell‐Based Therapy,” Advanced Materials 33 (2021): 2007379. 10.1002/adma.202007379 33629757

[31] N. Yin , Z. Zhang , Y. Ge , et al., “Polydopamine‐based Nanomedicines for Efficient Antiviral and Secondary Injury Protection Therapy,” Science advances 9 (2023): adf4098. 10.1126/sciadv.adf4098 PMC10266727 37315148

[32] H. Liu , X. Qu , H. Tan , et al., “Role of Polydopamine's Redox‐Activity on Its Pro‐Oxidant, Radical‐Scavenging, and Antimicrobial Activities,” Acta Biomaterialia 88 (2019): 181–196. 10.1016/j.actbio.2019.02.032 30818052

[33] Q. Hu , J. Li , T. Wang , X. Xu , Y. Duan , and Y. Jin , “Polyphenolic Nanoparticle‐Modified Probiotics for Microenvironment Remodeling and Targeted Therapy of Inflammatory Bowel Disease,” ACS Nano 18 (2024): 12917–12932. 10.1021/acsnano.4c00830 38720520

[34] Z. Ding , X. Bao , Y. Zhao , et al., “Polydopamine Nanodots Ameliorate Inflammatory Bowel Disease by Restoring Redox Homeostasis and Intestinal Microenvironment,” Advanced Science 12 (2025): 08674. 10.1002/advs.202508674 PMC12713062 40847778

[35] Y. Yu , Z. Huang , Y. Zhou , et al., “Facile and Highly Sensitive Photoelectrochemical Biosensing Platform Based on Hierarchical Architectured Polydopamine/Tungsten Oxide Nanocomposite Film,” Biosensors and Bioelectronics 126 (2019): 1–6. 10.1016/j.bios.2018.10.026 30388548

[36] T. Bedhiafi , S. Idoudi , A. A. Alhams , et al., “Applications of Polydopaminic Nanomaterials in Mucosal Drug Delivery,” Journal of Controlled Release 353 (2023): 842–849. 10.1016/j.jconrel.2022.12.037 36529384

[37] M. Ikram , S. Rasheed , A. M. Afzal , et al., “Ultrasensitive V Doped WO_3_ 1D Nanorods Heterojunction Photodetector With Pronounced Photosensing Activities,” Journal of Alloys and Compounds 909 (2022): 164753.

[38] L. Fan , J. Xie , Z. Zhang , Y. Zheng , D. Yao , and T. Li , “Magnetically Recoverable Fe_3_O_4_@Polydopamine Nanocomposite as an Excellent co‐catalyst for Fe^3+^ Reduction in Advanced Oxidation Processes,” Journal of Environmental Sciences 92 (2020): 69–78. 10.1016/j.jes.2020.02.006 32430134

[39] M. Vaghari‐Tabari , F. Alemi , M. Zokaei , et al., “Polyphenols and Inflammatory Bowel Disease: Natural Products with Therapeutic Effects?,” Critical Reviews in Food Science and Nutrition 64 (2024): 4155–4178. 10.1080/10408398.2022.2139222 36345891

[40] A. M. Casey , D. G. Ryan , H. A. Prag , et al., “Pro‐inflammatory Macrophages Produce Mitochondria‐Derived Superoxide by Reverse Electron Transport at Complex I That Regulates IL‐1β Release During NLRP3 Inflammasome Activation,” Nature Metabolism 7 (2025): 493–507. 10.1038/s42255-025-01224-x PMC11946910 39972217

[41] A. Gilliland , J. J. Chan , T. J. De Wolfe , H. Yang , and B. A. Vallance , “Pathobionts in Inflammatory Bowel Disease: Origins, Underlying Mechanisms, and Implications for Clinical Care,” Gastroenterology 166 (2024): 44–58. 10.1053/j.gastro.2023.09.019 37734419

[42] C. Yang and D. Merlin , “Unveiling Colitis: A Journey Through the Dextran Sodium Sulfate‐Induced Model,” Inflammatory Bowel Diseases 30 (2024): 844–853. 10.1093/ibd/izad312 PMC11063560 38280217

[43] W. Liang , G. Chen , B. Zheng , et al., “Biocompatible Nanozyme Shell‐Armed Probiotic With Inflammation Targeting and Scavenging Properties Enables Effective Treatment of Colitis,” Advanced Materials 38 (2025): 08532. 10.1002/adma.202508532 41190871

[44] X. Cao , S. Tao , W. Wang , et al., “Ternary Inulin Hydrogel With Long‐Term Intestinal Retention for Simultaneously Reversing IBD and Its Fibrotic Complication,” Nature Communications 15 (2024): 8428. 10.1038/s41467-024-52722-7 PMC11438902 39341804

[45] I. D. Iliev , A. N. Ananthakrishnan , and C. J. Guo , “Microbiota in Inflammatory Bowel Disease: Mechanisms of Disease and Therapeutic Opportunities,” Nature Reviews Microbiology 23 (2025): 509–524. 10.1038/s41579-025-01163-0 PMC12289240 40065181

[46] S. T. Radhakrishnan , B. H. Mullish , M. L. Olbei , et al., “Deciphering the Microbiome–Metabolome Landscape of An Inflammatory Bowel Disease Inception Cohort,” Gut Microbes 17 (2025): 2527863. 10.1080/19490976.2025.2527863 PMC12279270 40679059

[47] R. Huang , F. Wu , Q. Zhou , et al., “Lactobacillus and Intestinal Diseases: Mechanisms of Action and Clinical Applications,” Microbiological Research 260 (2022): 127019. 10.1016/j.micres.2022.127019 35421680

[48] X. Lin , T. Hu , Z. Wu , et al., “Isolation of Potentially Novel Species Expands the Genomic and Functional Diversity of Lachnospiraceae,” Imeta 3 (2024): 174. 10.1002/imt2.174 PMC11170972 38882499

[49] P. D. Cani , C. Depommier , M. Derrien , A. Everard , and W. M. de Vos , “Akkermansia Muciniphila: Paradigm for Next‐Generation Beneficial Microorganisms,” Nature Reviews Gastroenterology & Hepatology 19 (2022): 625–637. 10.1038/s41575-022-00631-9 35641786

[50] N. J. Mullen and P. K. Singh , “Nucleotide Metabolism: A Pan‐Cancer Metabolic Dependency,” Nature Reviews Cancer 23 (2023): 275–294. 10.1038/s41568-023-00557-7 PMC10041518 36973407

[51] M.‐X. Li , M.‐Y. Li , J.‐X. Lei , et al., “Huangqin Decoction Ameliorates DSS‐induced Ulcerative Colitis: Role of Gut Microbiota and Amino Acid Metabolism, mTOR Pathway and Intestinal Epithelial Barrier,” Phytomedicine 100 (2022): 154052. 10.1016/j.phymed.2022.154052 35344714

[52] S. Chitkara and G. E. Atilla‐Gokcumen , “Decoding Ceramide Function: How Localization Shapes Cellular Fate and How to Study It,” Trends in Biochemical Sciences 50 (2025): 356–367. 10.1016/j.tibs.2025.01.007 40000311

[53] C. R. Reiter , R. Rebiai , A. Kwak , et al., “The Pathogenic Sphingolipid Psychosine Is Secreted in Extracellular Vesicles in the Brain of a Mouse Model of Krabbe Disease,” ASN Neuro 14 (2022): 17590914221087817. 10.1177/17590914221087817 PMC8943320 35300522

[54] P. Jáuregui , “Microbiota Reprogramming of Macrophages,” Nature Immunology 26 (2025): 1839. 10.1038/s41590-025-02338-w 41162762

[55] H. Zhu , H. Guo , N. Sun , et al., “Faecalibaculum Rodentium Alleviates Ionizing Radiation‐Induced Damage in Mice by Improving Intestinal Integrity and Hematopoiesis via Its Metabolite Butyrate,” Advanced Science 13 (2025): 09383. 10.1002/advs.202509383 PMC12786293 41082369

[56] W. Qing , H. Chen , X. Ma , et al., “Gut Dysbiosis‐induced Vitamin B6 Metabolic Disorder Contributes to Chronic Stress‐related Abnormal Behaviors in a Cortisol‐independent Manner,” Gut Microbes 17 (2025): 2447824. 10.1080/19490976.2024.2447824 PMC11730634 39773070

[57] Z. Jin , Y. Yang , Y. Cao , et al., “The Gut Metabolite 3‐hydroxyphenylacetic Acid Rejuvenates Spermatogenic Dysfunction in Aged Mice Through GPX4‐mediated Ferroptosis,” Microbiome 11 (2023): 212. 10.1186/s40168-023-01659-y PMC10523725 37752615

[58] Y. Fu , D. V. Guzior , M. Okros , et al., “Balance Between Bile Acid Conjugation and Hydrolysis Activity Can Alter Outcomes of Gut Inflammation,” Nature Communications 16 (2025): 3434. 10.1038/s41467-025-58649-x PMC11985902 40210868

[59] R. Okumura and K. Takeda , “The Role of the Mucosal Barrier System in Maintaining Gut Symbiosis to Prevent Intestinal Inflammation,” Seminars in Immunopathology 47 (2024): 2. 10.1007/s00281-024-01026-5 PMC11599372 39589551

