# Supplementary material for: Nano‐Backpack Engineered Probiotics with Reactive Oxygen Species–Responsive Tungsten Release for Synergistic Therapy of Inflammatory Bowel Disease
Source: Adv Sci (Weinh). 2026 Jun 26;13(52):e76291. doi: 10.1002/advs.76291 (PMC13337005; doi:10.1002/advs.76291)
Supplement: Supplementary file 1 [file ADVS-13-e76291-s001.docx]

Supporting Information

**Nano-Backpack Engineered Probiotics with ROS-Responsive Tungsten Release for Synergistic Therapy of Inflammatory Bowel Disease**

‡Yang Yang, ‡Liucan Wang, ‡Guoqing Chen, Yuanling Zhang, Jiarui Shi, Wenzhe Fan, Min Yu*, Jixi Zhang*, Hua Yang*

Y. Yang, Prof. H. Yang*

Chongqing Medical University, Chongqing, 400016, China.

E-mail: huayang@cqu.edu.cn (Hua Yang)

Y. Yang, L. Wang, G. Chen, Y. Zhang, Prof. M. Yu*, Prof. H. Yang*

Department of General Surgery, Chongqing Academy of Medical Sciences, Chongqing General Hospital, Chongqing University, Chongqing, 401147, China.

E-mail: minyu@cqu.edu.cn (Min Yu)

L. Wang, J. Shi, W. Fan, Prof. J. Zhang*

Key Laboratory of Biorheological Science and Technology, Ministry of Education, College of Bioengineering, Chongqing University, Chongqing, 400044, China.

E-mail: jixizhang@cqu.edu.cn (Jixi Zhang)

‡ Y. Yang, L. Wang and G. Chen contributed equally to this work.

**Materials.** Unless otherwise noted, all reagent-grade chemicals were used as received, and distilled water was used for the preparation of all aqueous solutions. Tungsten trioxide (WO_3_ NPs, particle size <100 nm, and lipopolysaccharides (LPS) were obtained from Sigma-Aldrich (St. Louis, MO, USA). Dextran Sulfate Sodium (DSS, molecular weight 36,000-50,000 Da) was purchased from MP Biomedicals (Solon, OH, USA). PBS buffer, dopamine hydrochloride (DA), Cy5.5-NHS ester, Fluorescein isothiocyanate (FITC), Rhodamine B, 1,1-diphenyl-2-picrylhydrazyl (DPPH), and 2,2’-azino-bis (3-ethylbenzothiazoline-6-sulf- onic acid) diammonium salt (ABTS) were acquired from Aladdin Biochemical Technology Co., Ltd. (Shanghai, China). Chitosan (deacetylation degree >90%, viscosity <100 mPa·s), Tris(hydroxymethyl)aminomethane, LB broth, MacConkey broth, agar powder, and superoxide dismutase (SOD) activity assay kit were sourced from Solarbio Science & Technology Co., Ltd. (Beijing, China). Absolute ethanol, anhydrous methanol, and hydrogen peroxide (H_2_O_2_) were obtained from Chuandong Chemical Industry Co., Ltd. (Chongqing, China). 2’,7’-Dichlorodihydrofluorescein diacetate (DCFH-DA) was purchased from Heowns Biochemical Technology Co., Ltd. (Tianjin, China). Cell Counting Kit-8 (CCK-8) and Live/Dead Cell Staining Kit were acquired from Beyotime Biotechnology Co., Ltd. (Shanghai, China). Bacterial Dissimilatory Nitrate Reductase Activity Assay Kit was purchased from GenMed Scientifics (Santa Ana, CA, USA). Simulated gastric fluid (SGF), simulated intestinal fluid (SIF), simulated colonic fluid (SCF) were acquired from Yuanye Bio-Technology Co., Ltd. (Shanghai, China). Dihydroethidium (DHE) staining kit was purchased from Servicebio Technology Co., Ltd. (Wuhan, China). Antibodies against ZO-1(Cat No. 21773-1-AP), Occludin (Cat No. 27260-1-AP), and β-actin (Cat No. 20536-1-AP) were purchased from Proteintech (Wuhan, China), and the MUC2 antibody (Cat No. ab272692) was purchased from Abcam (Cambridge, UK). Antibodies against the inflammatory cytokines TNF-α (Cat No. AF7014), IL-6 (Cat No. DF6087), IL-1β (Cat No. AF5103), and IL-10 (Cat No. DF6894) were purchased from Affinity Biosciences (Changzhou, China).

**Strains and Culture Conditions.** The probiotic strain *Bacillus subtilis* (BS, ATCC 6051) used in this study was purchased from Bio-Sci Plasmid & Strain Resources Co., Ltd. (Hangzhou, China). A single colony of BS was picked from an LB agar plate and inoculated into 10 mL of LB liquid medium, followed by overnight incubation at 37°C with shaking at 200 rpm. For colony counting, cultures were spread on LB agar plates.

**Cells and animals.** HT-29 cells and RAW264.7 cells were acquired from Pricella Biotechnology Co., Ltd. (Wuhan, China). Cells were cultured in McCoy’s 5A medium or DMEM, supplemented with 10% fetal bovine serum (FBS) and 1% penicillin/streptomycin solution (100 U mL^-1^ penicillin and 100 µg mL^-1^ streptomycin) at 37°C in a humidified incubator with 5% CO₂.

Adult male C57BL/6 mice (6-8 weeks old) were provided by Hunan SJA Laboratory Animal Co., Ltd. (Changsha, China). All animals were housed under specific pathogen-free (SPF) conditions at 22-25°C and 50-60% relative humidity with a 12:12 h light-dark cycle. Food and water were provided *ad libitum*. The experimental procedures involving animals were approved by the Animal Ethics Committee of Chongqing University (Approval No. CQU-IACUC-RE-202205-001) and were performed in accordance with the institutional guidelines.

**Characterization.** TEM was performed using a JEM-2010 microscope (JEOL, Japan). SEM was conducted with a Regulus 8100 system (Hitachi, Japan). Zeta potential and hydrodynamic size were measured using a Zetasizer Nano ZS90 (Malvern Panalytical, UK). UV-Vis absorption spectra were recorded on a SPECORD 250 PLUS spectrometer (Analytik Jena, Germany). CLSM images were acquired with an LSM 510 META system (Zeiss, Germany). *In vivo* animal imaging was performed using an IVIS Lumina III platform (PerkinElmer, USA). Flow cytometry analysis was conducted on a BD LSRFortessa analyzer (Becton Dickinson, USA). Bacterial optical density (OD_600_) was measured with a Model 550 microplate reader (Bio-Rad, USA). Tungsten ions concentration was determined by ICP-MS using an Optima 8300 spectrometer (PerkinElmer, USA). Murine colonoscopy was performed using an IMAGE1 S endoscopic system (KARL STORZ, Germany).

**Bacterial Viability Assay.** 10 μL of BS or BSCS@WO₃@PDA suspension (bacterial concentration: 1 × 10^9^ CFU mL^-1^) was inoculated into LB broth and incubated in a shaker incubator at 37°C with 200 rpm agitation. To monitor bacterial growth, 100 μL aliquots of the culture were transferred to a 96-well microplate at hourly intervals. The optical density at 600 nm (OD_600_) was immediately measured using a microplate reader. The growth curves were plotted. Subsequently, samples were serially diluted, spread onto LB agar plates, and incubated overnight at 37°C for colony counting.

**CCK-8 assay**. HT-29 cells were seeded in 96-well plates (1×10^4^ cells/well) and incubated with various concentrations of WO_3_@PDA NPs, BS, or BSCS@WO_3_@PDA for 24 h. Subsequently, CCK-8 working solution (CCK-8 reagent: McCoy’s 5A medium = 1:9) was added to each well. After incubation at 37°C for 30 min, the absorbance at 450 nm (OD_450_) was measured using a microplate reader, and cell viability was calculated relative to untreated control cells.

**Live/Dead Cell Staining Assay.** HT-29 cells were seeded in 6-well plates (5×10^5^ cells/well) and cultured for 24 h. After washing with PBS, cells were treated with serum-free McCoy’s 5A medium containing 2 mM H_2_O_2_ and WO_3_@PDA NPs for 24 h. Cells were then stained with the Calcein-AM/PI working solution according to the manufacturer's protocol and imaged under a fluorescence microscope. (Calcein-AM, *Excitation:* 494 nm, *Emission: 517 nm*; PI, *Excitation:* 535 nm, *Emission:* 617 nm)

***In Vivo* Toxicity Assay.** Male C57BL/6 mice (6-8 weeks old, body weight 22-25 g) were acclimated for 7 days and divided into control and experimental groups (n = 5 per group). The experimental group received daily oral gavage of BSCS@WO_3_@PDA suspension (1 × 10^8^ CFU BS / 40 mg kg^-1^ WO_3_@PDA), while the control group received an equal volume of PBS for 7 consecutive days. Body weight was recorded daily. Mice were then euthanized on day 8. Blood was collected via retro-orbital puncture, allowed to clot for 2 h, and centrifuged at 1000 × g for 15 min to obtain serum. Serum biochemical parameters, including alanine aminotransferase (ALT), aspartate aminotransferase (AST), total bilirubin (TBIL), direct bilirubin (DBIL), albumin (ALB), urea nitrogen (UREA), and creatinine (CREA), were measured using an automatic biochemical analyzer. Major organs (heart, liver, spleen, lungs, kidneys) and the entire gastrointestinal tract (stomach, duodenum, jejunum, ileum, and colon) were harvested, fixed in 4% paraformaldehyde, embedded in paraffin, sectioned, and stained with hematoxylin and eosin (H&E) for microscopic examination.

**Histological Evaluation.** Colon tissue morphology was examined by H&E staining and scored for histopathological lesions according to established criteria (Table S3, Supporting Information). AB-PAS staining was used to evaluate mucin production. DHE staining was applied to assess oxidative stress, while IHC for MPO and inflammatory cytokines (TNF-α, IL-6, IL-1β, and IL-10) was conducted to evaluate tissue inflammation. The intestinal epithelial barrier function was assessed via IF staining for ZO-1 and Occludin.

**Western Blot Analysis.** Colon tissues were homogenized rapidly after euthanasia. Proteins were extracted, quantified, and separated by SDS-PAGE, and transferred to PVDF membranes. Membranes were blocked and incubated overnight at 4°C with primary antibodies against MUC2, ZO-1, and Occludin (dilution ratio: 1:1000). After washing, the membranes were incubated with appropriate HRP-conjugated secondary antibodies at room temperature for 1 h. Protein bands were visualized using SuperSignal West Pico PLUS Chemiluminescent Substrate and imaged.

**RT-qPCR.** Total RNA was extracted from RAW 264.7 cells and colon tissues using the isothiocyanate/phenol-chloroform method and isolated with TRIzol reagent (Servicebio Technology Co., Ltd., Wuhan, China). cDNA was reverse-transcribed from RNA using the PrimeScript RT reagent Kit (Takara Bio, Inc., Otsu, Japan). The amplified cDNA was used as template DNA for PCR assays with specific primers. The RT reaction was performed in a thermal cycler (Bio-Rad Laboratories, Inc., Hercules, CA, USA) at 42°C for 2 min, followed by 37°C for 15 min and 85°C for 5 sec. Subsequently, the mRNA expression levels of TNF-α, IL-1β, IL-6, and IL-10 were normalized to β-actin and calculated using the 2^(-ΔΔCt) method. Primer sequences are listed in Table S4 (Supporting Information).

**Table S1.** Size, PDI, zeta potential of the WO_3_ NPs and WO_3_@PDA NPs measured by DLS. Data are presented as mean ± standard deviation (SD) (n = 3).

| Nanoformulation | Size (nm) | PDI | Zeta potential (mV) |
| --- | --- | --- | --- |
| WO_3_ NPs | 158.4 ± 10.9 | 0.18 | -25.9 ± 0.2 |
| WO_3_@PDA NPs | 210.7 ± 13.2 | 0.19 | -22.9 ± 1.7 |

**Table S2.** The scoring system of the disease activity index (DAI).

| Score | Body weight loss | Stool consistency | Fecal occult blood |
| --- | --- | --- | --- |
| 0 | ≤1% | Normal | Negative |
| 1 | 1-5% | Soft stools | Trace blood |
| 2 | 5-10% | Mucoid stools | Visible blood in stool |
| 3 | 10-20% | Diarrhea | Gross rectal bleeding |
| 4 | ＞20% | Severe diarrhea with incontinence | Severe bleeding with anemia |

**Table S3.** Histological scoring system of the colon tissues.

| Score | Ulcer | Inflammatory infiltration | Epithelial changes |
| --- | --- | --- | --- |
| 0 | None | None | Normal |
| 1 | Small area | Crypt | Loss of goblet cell |
| 2 | Small- to medium- area | Muscularis mucosa | Large loss of goblet cells |
| 3 | Medium area | Universal infiltration in muscularis mucosa | Loss of crypt |
| 4 | Large area | Submucosa | Large loss of crypt or polypoid regeneration |

**Table S4.** Summary of gene-specific primer sequences.

| Gene name | Gene-specific primer sequences (5’ to 3’). |
| --- | --- |
| β-actin | Forward: ACTGTCGAGTCGCGTCCA |
|  | Reverse: TCATCCATGGCGAACTGGTG |
| TNF-α | Forward: GACGTGGAACTGGCAGAAGAG |
|  | Reverse: TTGGTGGTTTGTGAGTGTGAG |
| IL-1 β | Forward: TGCCACCTTTTGACAGTGATG |
|  | Reverse: TGATGTGCTGCTGCGAGATT |
| IL-6 | Forward: CCCCAATTTCCAATGCTCTCC |
|  | Reverse: CGCACTAGGTTTGCCGAGTA |
| IL-10 | Forward: CCAAGGTGTCTACAAGGCCA |
|  | Reverse: GCTCTGTCTAGGTCCTGGAGT |

**Figure S1.** Digital photograph of the dispersions of WO_3_ NPs and WO_3_@PDA NPs.

**Figure S2.** TEM image of WO_3_ NPs. Scale bar: 100 nm.

**Figure S3.** Hydrodynamic size distribution of (a) WO_3_ NPs and (b) WO_3_@PDA NPs measured by DLS.

**Figure S4.** HAADF-STEM and corresponding element mapping images of WO_3_@PDA NPs (W, O, C, N). Scale bar: 100 nm.

**Figure S5.** SEM images of BSCS@WO_3_@PDA_25_ (1 × 10^7^ CFU BS / 25 μg WO_3_@PDA), BSCS@WO_3_@PDA_50_ (1 × 10^7^ CFU BS / 50 μg WO_3_@PDA), BSCS@WO_3_@PDA_100_ (1 × 10^7^ CFU BS / 100 μg WO_3_@PDA), BSCS@WO_3_@PDA_200_ (1 × 10^7^ CFU BS / 200 μg WO_3_@PDA. Scale bar: 1 μm.

**Figure S6.** UV-Vis absorption spectra of WO_3_@PDA NPs, BS, BSCS and BSCS@WO_3_@PDA.

**Figure S7.** UV-vis absorption spectra of the scavenging reactions of (a) DPPH•, (b) •OH, (c) •O_2_⁻ , and (d) H_2_O_2_ by WO_3_@PDA NPs.

**Figure S8.** Radical scavenging evaluation of WO_3_@PDA NPs and BSCS@WO_3_@PDA toward (a) DPPH•, (b) •OH, (c) •O_2_^-^, and (d) H_2_O_2_ at a fixed WO_3_@PDA concentration of 200 μg mL^-1^, with the reaction visualized by color changes. Data are presented as mean ± standard deviation (SD) (n = 3); ns, not significant.

**Figure S9.** Relative expression levels of cytokine mRNA (TNF-α, IL-6, IL-1β, and IL-10) in RAW 264.7 cells after treatments of 100 μg mL^-1^ WO_3_@PDA NPs. Data are presented as mean ± SD (n = 3); ns, not significant.

**Figure S10.** (a) Representative photographs of bacterial colonies on LB agar plates after spread plating with BS, BSCS, and BSCS@WO_3_@PDA. (b) Corresponding bacterial survival rates. Data are presented as mean ± SD (n = 3); ns, not significant.

**Figure S11.** Bacterial colonies on LB agar plates following exposure of BS, BSCS, and BSCS@WO_3_@PDA after treatment with SGF, SIF, and H_2_O_2_ for varying durations.

**Figure S12.** (a) Schematic diagram of the *in vitro* simulated digestion process sequentially through SGF, SIF, and SCF. (b) Survival rates of BS and BSCS@WO_3_@PDA at predetermined time intervals during the simulated gastrointestinal transit. Data are presented as mean ± SD (n = 3).

**Figure S13.** Color change of WO_3_@PDA NPs after 24 h incubation with increasing concentrations of H_2_O_2_ (0 to 2.0 mM).

**Figure S14.** TEM images of WO_3_@PDA NPs following 24 h incubation with 1.0 mM H_2_O_2_.

**Figure S15.** (a) Representative plate images and (b) corresponding colony counts of *E. coli* on MacConkey agar after anaerobic co-incubation with BS, WO_3_@PDA NPs, and BSCS@WO_3_@PDA. Data are presented as mean ± SD (n = 3). ****p* < 0.001.

**Figure S16.** (a) Hemolysis rate of erythrocytes after treatment with WO_3_@PDA NPs at various concentrations. (b) Hemolysis rate of erythrocytes after treatment with WO_3_@PDA NPs, BS, and BSCS@WO_3_@PDA. Data are presented as mean ± SD (n = 3).

**Figure S17.** Cell viability of HT-29 Cells measured by the CCK-8 assay after 24 h co-incubation with varying concentrations of (a) WO_3_@PDA NPs, (b) BS , and (c) BSCS@WO_3_@PDA. (d) Comparison of the treatments at selected concentrations. Data are presented as mean ± SD (n = 3).

**Figure S18.** Optimization of the therapeutic dosage of BSCS@WO_3_@PDA in the DSS-induced colitis mice. (a) Schematic illustration of the experimental design for inducing colitis and the timeline for the oral administration of different dosages. (b) Body weight changes (%) relative to baseline. (c) Disease activity index (DAI) scores. The dosages are defined as Low (L, 0.5 × 10^8^ CFU BS / 20 mg kg^-1^ WO_3_@PDA), Medium (M, 1 × 10^8^ CFU BS / 40 mg kg^-1^ WO_3_@PDA), and High (H, 2 × 10^8^ CFU BS / 80 mg kg^-1^ WO_3_@PDA). Data are presented as mean ± SD (n = 5). ****p* < 0.001.

**Figure S19.** Representative photographs of the (a) perianal region and (b) excised colons. (c) Quantitative analysis of colon length. (d) Photographs of excised spleens. (e) Spleen index (spleen weight / body weight × 100%). (f) Histopathological scores of colon tissues. (g) Representative H&E staining of colon sections. Scale bars: 500 μm (overview), 100 μm (magnified). Data are presented as mean ± SD (n = 5). **P* < 0.05, ****P* < 0.001; ns, not significant.

**Figure S20.** Systemic biosafety assessment of BSCS@WO_3_@PDA (1 × 10^8^ CFU BS / 40 mg kg^-1^ WO_3_@PDA) in healthy C57BL/6 mice. (a) Schematic timeline of the oral administration experiment. (b) Body weight changes of mice monitored over 7 days. Data are presented as mean ± SD (n = 5).

**Figure S21.** Hematological and serum biochemical analyses reveal the systemic biosafety of BSCS@WO_3_@PDA in C57BL/6 mice. (a) Complete blood count parameters. (b) Key liver and kidney function indices. Data are presented as mean ± SD (n = 5); ns, not significant.

**Figure S22.** H&E-stained sections of the heart, liver, spleen, lung, and kidney of C57BL/6 mice from the control group and the BSCS@WO_3_@PDA-treated group. Scale bar: 100 μm.

**Figure S23.** Representative photographs of the gastrointestinal tract and H&E-stained sections of the stomach, duodenum, jejunum, ileum, and colon of C57BL/6 mice from the control group and the BSCS@WO_3_@PDA-treated group. Scale bar: 100 μm.

**Figure S24.** Representative immunofluorescence (IF) images and corresponding quantitative analysis of (a) F4/80-positive and (b) CD68-positive cells in colon tissue sections from the control group and the BSCS@WO_3_@PDA-treated group. Scale bar: 100 μm. Data are presented as mean ± SD (n = 5); ns, not significant.

**Figure S25**. (a) Representative immunohistochemistry (IHC) staining images for the expression of inflammatory cytokine proteins (TNF-α, IL-1β, IL-6, and IL-10) in colon tissues from the control group and the BSCS@WO_3_@PDA-treated group. Scale bar: 100 μm. (b) Quantitative analysis of the average optical density (AOD) for the corresponding cytokines. Data are presented as mean ± SD (n = 5); ns, not significant.

**Figure S26.** Fluorescence images of dissected organs (heart, liver, spleen, lung, kidney) from DSS-induced colitis mice after oral administration of Cy5.5-labeled BS, BSCS, or BSCS@WO_3_@PDA.

**Figure S27.** Bacterial colonies on LB agar plates of intestinal contents from DSS-induced colitis mice after oral administration of BS, BSCS, or BSCS@WO_3_@PDA.

**Figure S28.** (a) Representative IF images of F4/80-positive and CD68-positive cells in colon tissue sections from different groups. Scale bar: 100 μm. Relative fluorescence intensities of (b) F4/80-positive cells and (c) CD68-positive cells. Data are presented as mean ± SD (n = 5). **p* < 0.05, ****P* < 0.001; ns, not significant.

**Figure S29.** Quality assessment of amplified 16S rRNA V3–V4 regions from mouse intestinal content samples by agarose gel electrophoresis. Clear target bands between 400–500 bp indicate successful amplification and acceptable sample quality for subsequent sequencing.

**Figure S30.** Relative abundance of beneficial bacterial families: Muribaculaceae and Eggerthellaceae. Data are presented as mean ± SD (n = 5). ***P* < 0.01; ns, not significant.

**Figure S31.** Taxa listed according to their LDA scores determined from comparisons between the DSS and BSCS@WO_3_@PDA group using the LEfSe method. LDA (log10) > 3.0, *P* < 0.05 indicates a higher relative abundance in the corresponding group than that in another group.

**Figure S32.** Cladogram based on LEfSe analysis showing the community composition of the gut microbiota in mice. Taxa enriched in the DSS and BSCS@WO_3_@PDA group are highlighted in red or purple. Taxa without significant enrichment are shown in yellow. The LDA cutoff score was set at 3.0.

**Figure S33.** Quality control assessment of the metabolomics dataset. Principal component analysis (PCA) score plots in (a) negative and (b) positive ion mode. The plots show clear clustering of quality control (QC) samples and distinct separation trends among the Control, DSS, BSCS@WO_3_@PDA, and QC groups. (c, d) Hotelling’s T^2^ control charts for the sample sequence. All sample points, including QCs and experimental samples, fall within the 99% confidence limit (red dashed line), confirming high experimental reproducibility and instrumental stability over the entire run. (e, f) Distribution of the relative standard deviation (RSD) for metabolic features in QC samples. Over 80% of metabolic features exhibit an RSD below 30% in both ion modes, demonstrating that the data quality meets accepted standards for reliable statistical analysis.

**Figure S34.** Relative abundance of specific metabolites: (a) arginine*,* (b) cadaverine, (c) 3-Hydroxyphenylacetic acid, and (d) 2-Hydroxyphenylacetic acid. Data are presented as mean ± SD (n = 5). **P* < 0.01.

**Figure S35.** Visualization of therapeutic effects using an endoscopic video system for mice after being anesthetized by inhalation of isoflurane to visualize the therapeutic effect.

**Figure S36.** Western blot analysis of MUC2 and tight junction proteins (ZO-1 and Occludin) expression in colonic mucosa, with β-actin as control.

**Figure S37.** (a) Hematological and (b) serum biochemical analyses of healthy and DSS-induced colitis mice after treatment of BS, WO_3_@PDA NPs, or BSCS@WO_3_@PDA. Data are presented as mean ± SD (n = 5). **P* < 0. 05, ***P* < 0.01, ****P* < 0.001; ns, not significant.

**Figure S38.** H&E-stained sections of major organs (heart, liver, spleen, lung, kidney) from healthy and DSS-induced colitis mice after treatment of BS, WO_3_@PDA NPs, or BSCS@WO_3_@PDA. Scale bar: 100 μm.
